# A cytotoxic-skewed immune set point predicts low neutralizing antibody levels after Zika virus infection

**DOI:** 10.1016/j.celrep.2022.110815

**Published:** 2022-05-17

**Authors:** Elizabeth E. McCarthy, Pamela M. Odorizzi, Emma Lutz, Carolyn P. Smullin, Iliana Tenvooren, Mars Stone, Graham Simmons, Peter W. Hunt, Margaret E. Feeney, Philip J. Norris, Michael P. Busch, Matthew H. Spitzer, Rachel L. Rutishauser

**Affiliations:** 1 Departments of Otolaryngology-Head and Neck Surgery and Microbiology and Immunology, University of California San Francisco, San Francisco, CA 94143, USA; 2 Department of Medicine, Zuckerberg San Francisco General Hospital, University of California San Francisco, San Francisco, CA 94110, USA; 3 Vitalant Research Institute, San Francisco, CA 94104, USA; 4 Department of Laboratory Medicine, University of California San Francisco, San Francisco, CA 94143, USA; 5 Department of Pediatrics, University of California San Francisco, San Francisco, CA 94110, USA; 6 Gladstone-UCSF Institute for Genomic Immunology, San Francisco, CA 94158, USA; 7 Parker Institute for Cancer Immunotherapy, San Francisco, CA 94143, USA; 8 Chan Zuckerberg Biohub, San Francisco, CA 94158, USA; 9 Present address: Gilead Sciences, Inc., Foster City, CA 94404, USA; 10 These authors contributed equally; 11 Lead contact

## Abstract

Although generating high neutralizing antibody levels is a key component of protective immunity after acute viral infection or vaccination, little is known about why some individuals generate high versus low neutralizing antibody titers. Here, we leverage the high-dimensional single-cell profiling capacity of mass cytometry to characterize the longitudinal cellular immune response to Zika virus (ZIKV) infection in viremic blood donors in Puerto Rico. During acute ZIKV infection, we identify widely coordinated responses across innate and adaptive immune cell lineages. High frequencies of multiple activated cell types during acute infection are associated with high titers of ZIKV neutralizing antibodies 6 months post-infection, while stable immune features suggesting a cytotoxic-skewed immune set point are associated with low titers. Our study offers insight into the coordination of immune responses and identifies candidate cellular biomarkers that may offer predictive value in vaccine efficacy trials aimed at inducing high levels of antiviral neutralizing antibodies.

## In brief

McCarthy et al. use mass cytometry to longitudinally characterize peripheral cellular immune features during Zika virus (ZIKV) infection in non-pregnant adults. They identify distinct cellular immune signatures during acute infection that reliably predict the persistence of high versus low ZIKV-specific neutralizing antibody levels 6 months after infection.

## Graphical Abstract

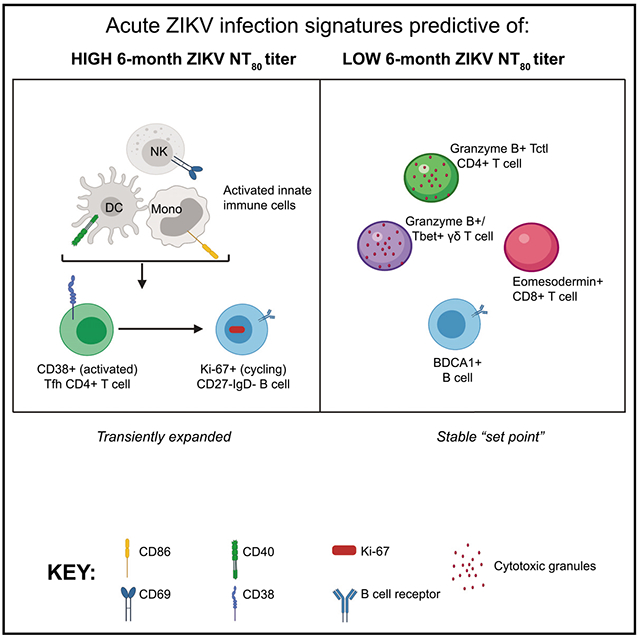

## INTRODUCTION

Infection of pregnant individuals with Zika virus (ZIKV), a flavivirus primarily transmitted to humans via the bite of an infected mosquito, can lead to persistent viral replication in the placenta and fetal brain that is associated with devastating fetal neurologic outcomes (Bhatnagar et al., 2017; Honein et al., 2017; Nithiyanantham and Badawi, 2019; Zorrilla et al., 2017). In contrast, for the majority of non-pregnant immunocompetent adults, ZIKV virus is rapidly cleared from the plasma (Barzon et al., 2018; Calvet et al., 2018; Coffey et al., 2017; Osuna et al., 2016; Stone et al., 2020) and infection is accompanied by mild symptoms such as fever, rash, and joint pain or can be asymptomatic (Lazear and Diamond, 2016; Rodriguez-Barraquer et al., 2019). Since the recent 2015–2016 epidemic in the Americas, there has been a considerable effort towards the development of a ZIKV vaccine, particularly for the prevention of mother-to-child transmission of infection (Abbink et al., 2018; Richner and Diamond, 2018; Shan et al., 2018). The majority of ZIKV vaccine candidates aim to induce durable, high-titer neutralizing antibody responses, which confer protection in animal models (Abbink et al., 2017; Ngono and Shresta, 2018).

Natural infection with ZIKV in humans generates robust ZIKV-specific antibody responses (Larocca et al., 2016; Rodriguez-Barraquer et al., 2019); however, there is wide inter-individual variation in the levels of ZIKV-specific antibodies that persist in the serum (Andrade et al., 2019; Rodriguez-Barraquer et al., 2019). Immunity to subsequent infection with ZIKV is likely to be influenced by the magnitude and durability of the ZIKV neutralizing antibody response (Abbink et al., 2016; Barouch et al., 2017; Larocca et al., 2016), but little is known about the factors that contribute to inter-individual variation in antibody responses. There is substantial cross-reactivity between virus-specific antibodies (Andrade et al., 2019; Dejnirattisai et al., 2016; Priyamvada et al., 2016) and T cell responses (Grifoni et al., 2017; Lim et al., 2018; Wen et al., 2017) generated after infection with ZIKV and those from the closely related and often co-circulating dengue virus (DENV). However, prior DENV exposure alone does not appear to explain the wide range of ZIKV antibody titers observed after natural infection (Andrade et al., 2019).

For other pathogens, baseline immune characteristics and/or signatures of early immune responses acutely after infection or vaccination have been shown to correlate with the magnitude of pathogen-specific antibody titers (Hu et al., 2019; Koutsakos et al., 2021; Li et al., 2017; Nakaya et al., 2015; Popper et al., 2018; Querec et al., 2009; Tan et al., 2014; Tsang et al., 2014). Some aspects of the innate cytokine and cellular immune responses to ZIKV infection have been described in humans (Barros et al., 2018; Cimini et al., 2017; Grifoni et al., 2018; Lai et al., 2018; Lum et al., 2018; Michlmayr et al., 2017; da Silva et al., 2019). However, the relationship between the acute-phase immune response and the generation of ZIKV-specific antibodies has not been characterized. This is in part due to the inherent challenges in identifying and establishing longitudinal cohorts of individuals identified during the earliest days of the acute phase of a natural infection.

Here, we used high-dimensional single-cell profiling with mass cytometry (cytometry by time of flight [CyTOF]) to deeply characterize the cellular innate and adaptive immune response during acute and convalescent ZIKV infection. We evaluated longitudinal peripheral blood samples collected from 25 individuals in a natural history cohort of healthy, non-pregnant adults from Puerto Rico who were found to be viremic with ZIKV at the time of blood donation during the recent ZIKV epidemic of 2015–2016 (Musso et al., 2017; Stone et al., 2020; Williamson et al., 2020). We found broadly coordinated cellular responses across immune cell lineages during acute ZIKV infection and identified distinct cellular immune signatures during acute ZIKV infection that were associated with the development and persistence of low versus high neutralizing antibody titers. In addition, we identified stable immune features that comprise a cytotoxic immune set point associated with low neutralizing antibody titers. Future vaccine efficacy trials for ZIKV and other acute viral infections may benefit from the inclusion of these candidate cellular biomarkers to aid in the prediction of neutralizing antibody titers, and additional strategies may be required to elicit stronger antibody responses in individuals with cytotoxic-skewed baseline immune set points.

## RESULTS

### Identifying immune cell populations that respond to acute ZIKV infection

**Figure 1.**
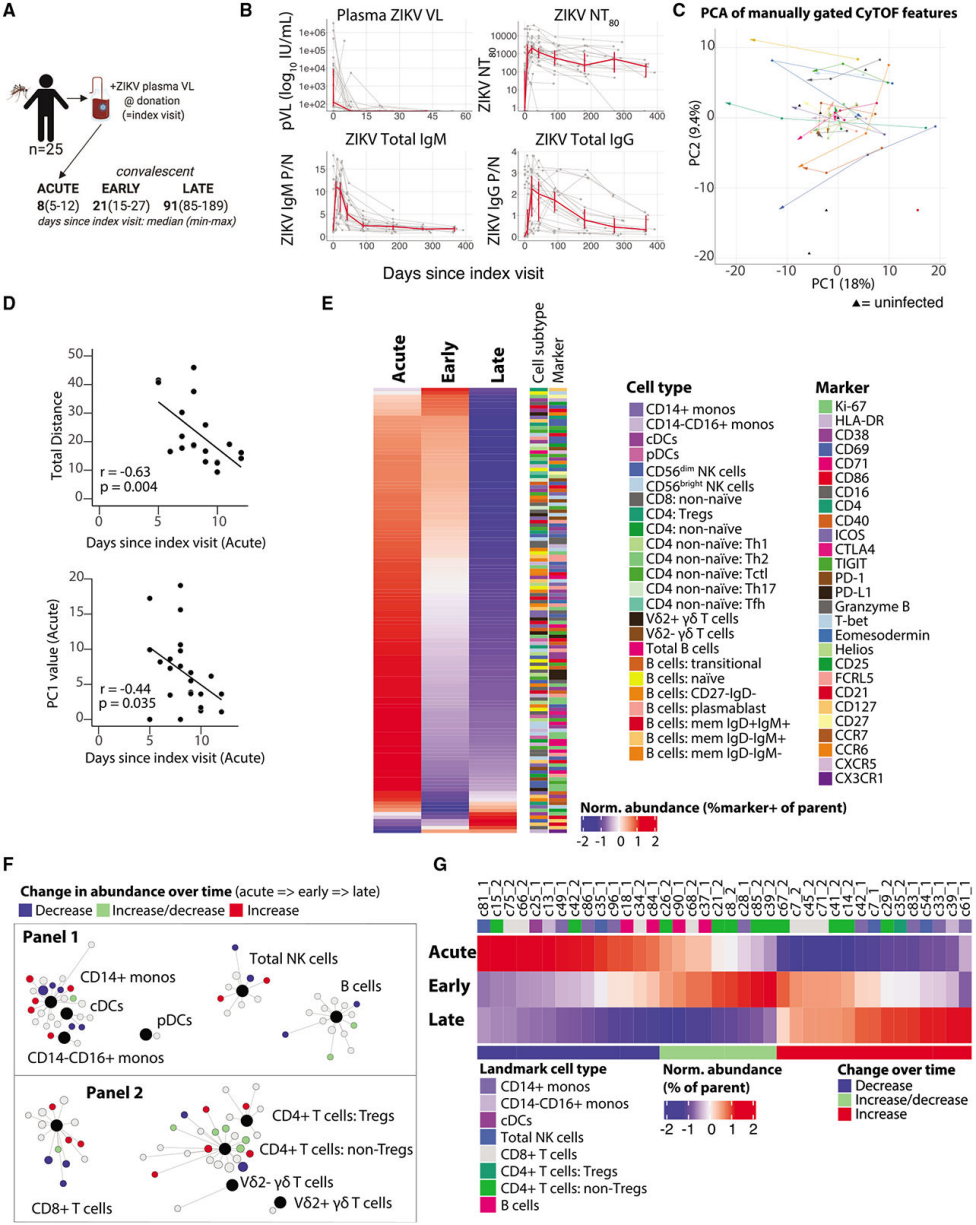
Acute infection with ZIKV elicits profound phenotypic changes across peripheral blood cellular immune populations (A) Twenty-five adults, viremic with acute ZIKV infection at the time of blood donation (index visit), had peripheral-blood sampling at up to three time points: acute phase of infection and early and/or late convalescence (see Table S1 for clinical characteristics). (B) Plasma ZIKV viral load (VL), neutralizing antibody titers (NT_80_) and total IgG and IgM levels of study-cohort participants. Red line connects median values at each sampling timepoint (±95% confidence interval [CI]). (C) Directed line plots for each participant in PCA space from early to later time points. Black triangles denote 8 uninfected control samples. (D) Scatterplots of days since index visit at the acute time point and the value of PC1 at the acute time point or the total distance traveled in PCA space between the acute and late convalescent time points (Spearman’s correlation with regression line). (E) Heatmap showing the *Z* score-normalized frequency of the log-adjusted feature abundances for the manually gated phenotypic features that change significantly over time (see Table S2 for list of features assessed). (F) SCAFFoLD maps showing clusters of cells associated with landmark cell-population nodes (black dots). Clusters that significantly change in abundance between the acute and late convalescent time points are labeled: increase (red), decrease (blue), or increase and then decrease (green). (G) Heatmap showing the normalized abundance of the clusters (Z score based on percentage of parent-cell-type population) that change significantly. Significance in (E)–(G) based on linear mixed effects (LME) model fit with p_adj < 0.05. See also Figure S1 and Tables S1 and S2

To characterize the cellular immune response to acute ZIKV infection, we designed two CyTOF antibody panels to phenotype innate immune and B cell (panel 1) and T cell (panel 2) features (see STAR Methods). We used these panels to analyze peripheral blood mononuclear cells (PBMCs) collected longitudinally at up to three time points during acute, early, and late convalescent phases of infection from 25 otherwise healthy blood donors in Puerto Rico who were found to be viremic for ZIKV at the time of blood donation (“index visit”; study participants are part of a larger REDS-III cohort; Figure 1A; Table S1; Data S1). Of the participants, 28% (7 of 25) were female, and the median age was 45 years (range 21–71). All participants mounted a detectable ZIKV immunoglobulin M (IgM), IgG, and neutralizing antibody response (reported as the 80% neutralization titers [NT_80_]; Figure 1B). Although all participants were viremic at the index visit, 68% (17 of 25) had not yet formed ZIKV-specific IgM responses. Of the participants with a collection visit at the first (“acute”) PBMC collection time point (median of 8 days after index), 100% had formed IgM antibodies and 22% (5 of 23) had residual detectable plasma viremia. There was substantial variation in both peak neutralizing antibody titers (ZIKV NT_80_ titers: 84–37,872) and follow-up titers 6 months after the index visit (0–6,286).

We first characterized how acute ZIKV infection perturbs the frequency and activation of different immune cell types in peripheral blood. We manually gated major landmark immune cell populations defined by standard lineage markers (e.g., classical [CD14+] monocytes, non-classical [CD14−CD16+] monocytes, plasmacytoid dendritic cells [pDCs], classical DCs [cDCs], CD56^bright/dim^ natural killer [NK] cells, CD4+ T cells, CD8+ T cells, B cells, etc.) and classically defined adaptive immune cell subsets (see Figure S1 for gating strategy and STAR Methods for mass-cytometry antibodies). We first evaluated the relative abundance of 40 cell types (landmark populations and adaptive immune subsets). We then evaluated the Boolean expression of 30 different phenotypic surface and intracellular proteins on these parent cell types, which yielded a total of 286 unique phenotypic features (see Table S2 for phenotypic features and Data S2 for all raw data from manual gating).

To broadly determine how the immune state is perturbed in the context of ZIKV infection, we performed principal-component analysis (PCA) on the manually gated CyTOF features (adjusted for age and sex). We mapped the trajectories across the three time points in PCA space for each individual ZIKV-infected participant (Figure 1C) as well as 8 control ZIKV-uninfected blood donors (black triangles). While there was variation between individuals, most participants followed a similar general trajectory from right to left along PC1 as they progressed from acute to convalescent ZIKV infection (not observed across longitudinal sampling of 6 separate ZIKV-uninfected individuals; Figure S1D). The number of days between the index and the acute time point negatively correlated with the total distance travelled in PCA space across the top five PCs as well as the value of PC1 at the acute time point (Figure 1D). These correlations suggest that both the PC1 coordinate and the distance travelled correspond to movement in virtual infection space as participants resolve their ZIKV infection.

To understand which cellular features contributed to this coordinated movement over time, we used linear mixed effect modelling on the age- and sex-adjusted feature abundances (see Data S3 for tables of all statistical-analysis results). While the frequency of most major immune cell types did not change significantly across the three sampled time points (Figures S2A and S2B), 128 of the 286 phenotypic features did change significantly across the three sampled time points (adjusted p value [p_adj] < 0.05; Figure 1E). The vast majority (95%) of these changing features were elevated at the acute time point and decreased in abundance by the late convalescent time point. A subset of these features initially remained elevated at the early convalescent time point, while others decreased sharply between the acute and early convalescence stage (e.g., most populations expressing Ki-67 and CD71).

To leverage the richness of our high-dimensional single-cell dataset, we performed unsupervised clustering using the SCAFFoLD algorithm that we have described previously, which associates cell clusters with user-defined landmark populations (Spitzer et al., 2015, 2017). We observed high concordance in the frequency of the pre-defined landmark immune cell populations between our manual gating and SCAFFoLD approaches (Figures S2C and S2D). Linear mixed effect modelling demonstrated that 15 of 34 clusters assigned to innate immune cell types and 23 of 56 clusters assigned to adaptive immune cell types (innate immune and B cell clusters from CyTOF panel 1, T cell clusters from panel 2) changed significantly in abundance as a percentage of their parent landmark population over time (Figures 1F and 1G; full raw and statistical analysis data available in Data S2 and S3). We again observed diversity in the direction and speed with which clusters changed in abundance over the three time points.

### Innate immune cell activation in acute ZIKV infection

**Figure 2.**
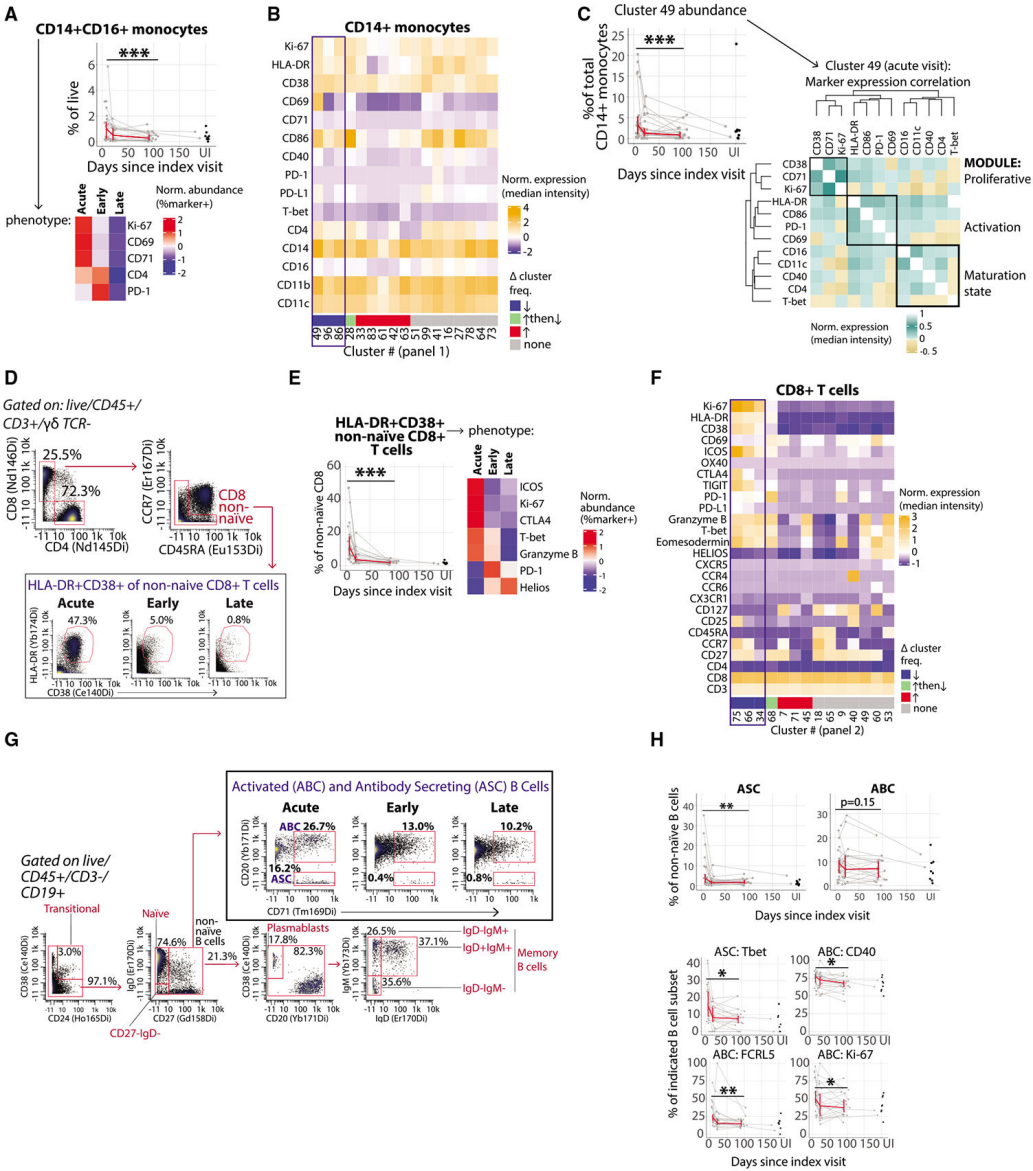
Transient accumulation of activated immune cells during acute ZIKV infection (A) Frequency (as a percentage of total live cells) and phenotype (*Z* scored proportion of cells that express each marker) of CD14+CD16+ monocytes across the course of acute and resolving ZIKV infection. (B) Heatmap showing *Z* score-normalized median expression of indicated markers (rows) for each monocyte-associated cell cluster (columns). Column annotation indicates clusters that significantly decrease (blue), increase (red), increase and then decrease (green), or remain unchanged (gray) in abundance (as a percentage of CD14+ monocytes; p_adj < 0.05). (C) Change in abundance of CD14+ monocyte cluster 49 (as a percentage of CD14+ monocytes; p_adj = 0.0002; left) and Spearman’s correlation matrix of marker expression on single cells in CD14+ monocyte cluster 49 from acute-visit samples (right). (D) Gating scheme for non-naïve CD8+ T cells that co-express HLA-DR and CD38. Percentages shown are the percentage of parent populations in plotted sample. (E) Frequency (as a percentage of non-naïve CD8+ T cells) and phenotype (Z scored proportion of cells that express each marker) of HLA-DR+CD38+ non-naïve CD8+ T cells across the course of acute and resolving ZIKV infection. (F) Phenotype (*Z* scored median expression of each marker) of CD8+ T cell clusters that significantly decrease (blue), increase (red), increase and then decrease (green), or remain unchanged (gray) in abundance. (G) Gating scheme for B cell subsets, including activated and antibody-secreting B cells (ABCs and ASCs, respectively). Percentages shown are the percentage of parent populations in the plotted sample. (H) Frequency (as a percentage of non-naïve B cells) and phenotype of ABCs and ASCs across the course of acute and resolving ZIKV infection; p_adj < 0.05). *p_adj < 0.05, **p_adj < 0.01, and ***p_adj < 0.001. (A, C, E, and H) Red line connects median values at each sampling time point (±95% CI). UI, uninfected. N = 25 participants. See also Figures S3-S5.

Little is known about the innate immune response to acute ZIKV infection in humans. Intermediate (CD14+CD16+) monocytes have been shown to increase in the peripheral blood of children with acute infection and are themselves a major target for ZIKV infection (Michlmayr et al., 2017, 2020). In adults, we also observed a transiently elevated level of intermediate monocytes during acute ZIKV infection (Figure 2A; see Figure S1 for gating). Intermediate monocytes in acute infection expressed higher levels of activation markers (Figure 2A). Manual gating and unsupervised clustering analyses revealed that acute infection was also associated with activation in the broader classical (CD14+) monocyte population (which includes CD14+CD16+ intermediate monocytes; Figure 2B) as well as non-classical (CD14−CD16+) monocytes (Figure S3A).

To understand how the expression of activation markers was coordinated on monocyte populations transiently increased in acute infection, we used our clustering analysis to investigate co-expression on individual cells contained within the classical CD14+ monocyte cluster (cluster 49) with the greatest median relative change in frequency (−75%) from the acute to late convalescent timepoints (Figure 2C). This revealed three modules of markers with coordinated expression patterns: (1) a proliferative module (Ki-67, CD71, and CD38), (2) an early activation module (HLA-DR, CD86, PD-1, and CD69), and (3) a monocyte maturation/differentiation module (CD16, CD11c, CD40, and CD4; Figure 2C). Thus, with unsupervised analysis, we identified modules representing distinct activation/differentiation states of transiently expanded monocytes.

The proportion of activated cDCs and pDCs was also increased in acute infection, and several activation markers were co-expressed on the cDC cluster with the greatest relative decrease in abundance as infection resolved (Figure S3B). Among NK cells, acute infection was associated with increased proliferation and activation in both CD56^bright^ and the more cytotoxic CD56^dim^ NK cells, which resolved during convalescence (Figure S3C). Collectively, these data demonstrate that acute ZIKV infection is characterized by the activation and differentiation of diverse innate immune cells.

### Accumulation of activated T and B cells in acute ZIKV infection

The population of HLA-DR+CD38+ CD8+ T cells has been found to be enriched for antigen-specific CD8+ T cells in other acute infections (Chandele et al., 2016; Mathew et al., 2020; Wang et al., 2018). Acute ZIKV infection was accompanied by a profound accumulation of cycling, activated non-naïve CD8+ T cells co-expressing HLA-DR and CD38 (Figures 2D and 2E). Indeed, our clustering analysis revealed that the expression of multiple activation markers on CD8+ T cells in acute ZIKV infection was tightly co-regulated on small sub-populations. The majority of HLA-DR+CD38+ CD8+ T cells were contained within three clusters of CD8+ T cells that expressed the highest levels of other activation markers (e.g., Ki-67, ICOS, CTLA-4, TIGIT, CD25) and were only transiently increased in acute infection (summed median frequency of c75, 66, 34 across time: 7.1% → 3.1% → 1.6%; Figure 2F). Acute ZIKV infection was also associated with a transient increase in the abundance of small sub-populations of cycling and activated non-naïve CD4+ T cell subsets and cytotoxic-skewed γδ T cells (Figure S4).

Using two established and correlated (Figure S5A) methods for identifying B cell populations that are actively secreting antibodies (CD38^hi^CD20^neg^ plasmablasts and CD71^hi^CD20^neg^ antibody-secreting cells [ASCs] [Ellebedy et al., 2016]), we noted a significant decrease in the frequency of these cells between acute infection and early/late convalescence (Figures 2G and 2H). Phenotypically, a larger proportion of ASCs and other B cell subsets expressed the transcription factor T-bet, which has been associated with B cell responses to viral infections (Johnson et al., 2020), during acute infection compared with early and late convalescence (p_adj = 0.02; Figures 2H and S5C).

An increased frequency of CD20^hi^CD71^hi^ activated B cells (ABCs) has also been described in other acute infections in humans (Ellebedy et al., 2016; Sutton et al., 2021). We observed a significant decrease over time in the proportion of ABCs expressing Ki-67, FCRL5, and CD40 (Figure 2H). Overall, multiple subsets of B cells transiently expressed several activation makers in acute ZIKV infection (Figures S5B and S5C). Finally, the expression of CD21 was lower on the two IgD-memory B cell populations during acute ZIKV infection (Figure S5C), which may identify cells recently exited from a germinal center (Lau et al., 2017).

### Coordinated activation of innate and adaptive immune cells in acute ZIKV infection

**Figure 3.**
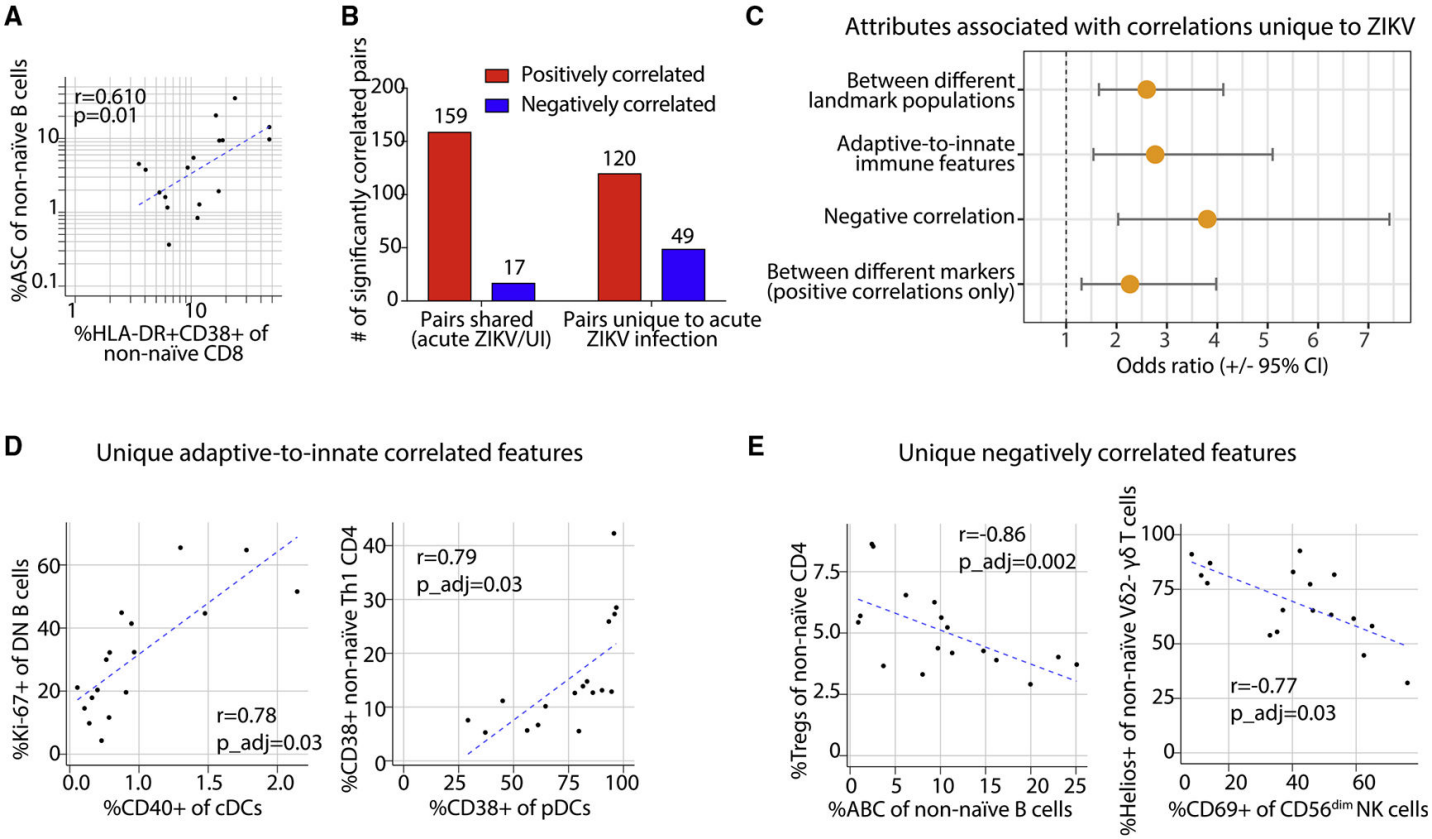
Coordinated activation across different cell types in acute ZIKV infection (A) Scatterplot of the frequency of ASC B cells and CD38+HLA-DR+ CD8+ T cells in acute ZIKV infection with regression line. (B) Number of significant (p_adj < 0.05) positive and negative correlations between cellular immune features that are present in acute ZIKV infection, grouped by those that are unique to ZIKV versus those shared with the uninfected (UI) cohort. (C) Odds ratio (±95% CI) that cellular immune feature correlations unique to ZIKV infection are more likely to be associated with different correlation attributes (compared with the correlations shared with the UI cohort). (D and E) Correlation plots of select features uniquely correlated in acute ZIKV infection (Spearman’s r with correlation line): (D) adaptive-to-innate immune features and (E) negatively correlated features. N = 17 participants (anti-ZIKV IgM- at index visit).

We next asked how these immune parameters during acute infection were coordinated to understand the intercellular dynamics that mediate the observed active immune response. We focused on the acute time point from 17 individuals without detectable anti-ZIKV IgM at their index visit to limit the variation in the data collected from participants, who may have been sampled at a different number of days following infection. We first interrogated features enriched for antigen-specific cells and found that the frequencies of ASC B cells and CD38+HLA-DR+ non-naïve CD8+ T cells were positively correlated (Spearman’s r = 0.61, p = 0.01; Figure 3A).

We broadly characterized the relationships of the acute-phase cellular features by computing correlations between all cellular features at the acute time point, revealing 279 feature pairs that were positively correlated and 66 that were negatively correlated during acute ZIKV infection (p_adj < 0.05). To focus on the correlations that were exclusive to acute ZIKV infection, we separated the feature pairs that were uniquely correlated during acute ZIKV infection, designated as “unique” (n = 169), from the remainder-labeled as “shared”—that were correlated both during acute infection as well as in the uninfected samples (n = 176; Figure 3B). Compared with the shared correlations, the unique correlations during acute ZIKV infection were more likely to be between features from different major landmark populations (odds of correlations being between features belonging to different/the same landmark populations among unique [105/64] versus shared [68/108] correlations; odds ratio [OR] 2.60 [95% confidence interval (CI): 1.65–4.12]; Figure 3C). The unique correlations were also more likely to be between (rather than within) adaptive and innate immune features (OR 2.77 [1.54–5.10]). Thus, during acute ZIKV infection, there was more coordination across arms of the immune system (e.g., significant unique correlation between CD38+ pDCs and CD38+ Th1 CD4+ T cells [r = 0.79, p_adj = 0.03]; Figure 3D). We also found that within the positive correlations, the unique correlations were more likely to be between different markers (OR 2.27 [1.31–3.98]; e.g., CD40+ cDCs and Ki-67+ double-negative [DN; CD27-IgD-] B cells [r = 0.78, p_adj = 0.03]; Figure 3D). Together, these findings suggest that during acute ZIKV infection, there is broad coordination of the expression of a diversity of activation markers across adaptive and innate immune cell types.

### Individuals exhibit inversely correlated cellular immune signatures during acute ZIKV infection

**Figure 4.**
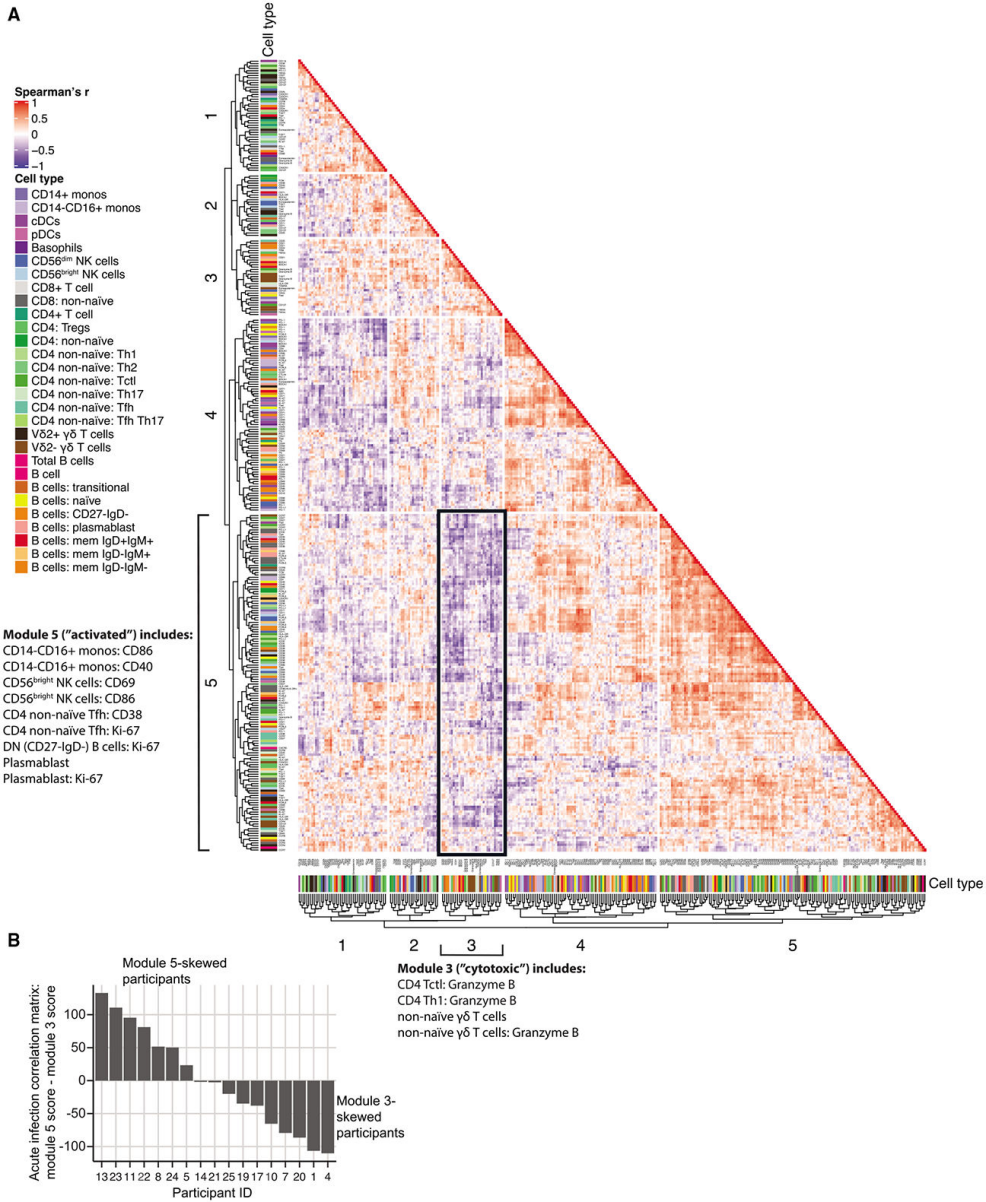
Correlated immune cell features during acute ZIKV infection (A) Correlation heatmap depicting Spearman’s correlation values (no significance cutoff) of all manually gated features from acute ZIKV infection, representing the 17 participants who were ZIKV IgM- (“pre-IgM”) at the index visit. Hierarchical clustering was used to group cellular features into five modules. Negatively correlated modules 3 and 5 are indicated with bold outlines. (B) Distribution of (module 5 score - module 3 score) values at the acute visit among the pre-IgM study participants. N = 17 participants (anti-ZIKV IgM- at index visit). See also Figure S5.

We next asked if there were inter-individual differences between study participants in acute ZIKV infection that may help to explain the large differences in neutralizing antibody titers. Indeed, the feature pairs uniquely correlated during acute infection were more likely to be negatively correlated across study participants compared with those shared with the uninfected state (OR 3.80 [2.03–7.42]; Figures 3B and 3C). For example, uniquely in acute ZIKV infection, we observed negative correlations between the frequency of ABCs and CD4+ regulatory T cells (Tregs) (r = −0.86, p_adj = 0.002) and between CD69+ CD56^dim^ NK cells and Helios+ Vδ2- γδ T cells (r = −0.77, p_adj = 0.03; Figure 3E). We hypothesized that these negatively correlated features unique to acute infection reflected inter-individual variability in the acute-phase immune response. To investigate, we performed hierarchical clustering of the acute-infection-feature correlation matrix (Figure 4A), which revealed the presence of two modules (modules 3 and 5) that contained sets of features which were inversely correlated with one another (average correlation: r = −0.79; see Data S2 for features contained within each module). While the module 5 immune signature was enriched for features that represent activated innate and adaptive immune cell types, 54% of which were transiently elevated in acute infection, the module 3 signature was enriched for features that reflect more cytotoxic-differentiated cell types, 91% of which were “stable,” meaning they did not change in abundance in the context of acute ZIKV infection. During acute infection, some individuals had a higher module 5 score, reflecting dynamic immune activation during acute ZIKV infection, while others had a higher module 3 score, suggesting a more cytotoxic-differentiated immune state (Figure 4B). To determine the clinical significance of these acute-phase signatures, we next asked if these distinct acute-phase cellular immune signatures could predict the magnitude of ZIKV neutralizing antibody responses after infection.

### Transient expansion of activated cell types in acute infection predicts high neutralizing antibody titers after ZIKV infection

**Figure 5.**
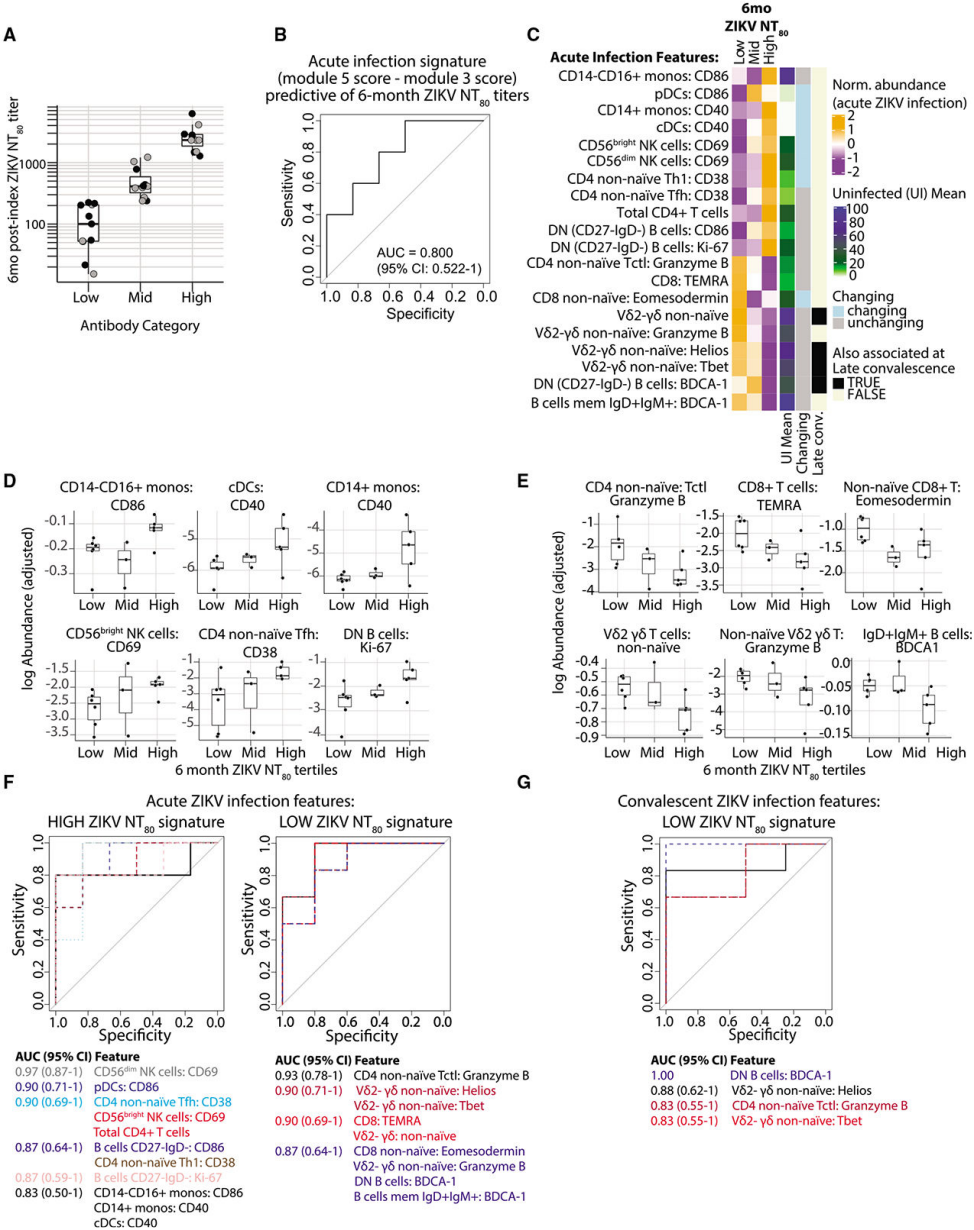
Distinct cellular immune signatures are associated with the development of high versus low ZIKV neutralizing antibody titers 6 months after infection (A) ZIKV neutralizing antibody titers (NT_80_) measured approximately 6 months post-index visit in the overall REDS-III study participants (gray dots) and the sub-cohort studied here (black dots). Participants were divided into tertiles based on these values. (B) Receiver operating characteristic (ROC) curve for predicting high- versus low-titer individuals using the difference between the acute-phase module 5 and 3 signature scores. (C) Heatmap showing *Z* score-normalized abundance at the acute visit for cellular features that were significantly (p_adj < 0.05) increased in high versus low 6-month NT_80_ titer participants at the acute time point. Row annotations for each feature indicate the following: mean values in a cross-sectional UI control cohort, whether or not the abundance of the feature significantly changed across time between acute to convalescent infection, and whether or not the abundance of the feature was also present at a significantly higher frequency (p_adj < 0.05) in the same group (high-versus low-titer participants) at the late convalescent time point. (D and E) Abundance (log-adjusted) of features during acute ZIKV infection associated with high (D) versus low (E) 6-month neutralizing antibody titers. (F) ROC curves for predicting high- versus low-titer individuals using the acute ZIKV cellular features from (C) that are associated with high (left) versus low (right) 6-month ZIKV NT_80_ titers. (G) ROC curves for predicting high-versus low-titer individuals using the late convalescent features associated with low 6-month ZIKV neutralizing antibody titers. (B, F, and G) The area under the curve (AUC) value and 95% CI for the features corresponding to each curve are colored by AUC value for each plot. N = 14 participants with 6-month ZIKV NT_80_ titer data available (anti-ZIKV IgM- at index visit).

We observed a large range in the titers of ZIKV neutralizing antibodies (NT_80_) that persisted several months after the resolution of acute infection (Figure 1B). In order to identify the cellular immune features during acute ZIKV infection that associated with the development of a high versus low ZIKV NT_80_ titer 6 months after infection, we again focused our analysis on individuals sampled as early as possible in the course of infection (i.e., who were ZIKV IgM- at the index visit). Since prior exposure to DENV is associated with significantly higher long-term ZIKV NT_80_ antibody titers (Rodriguez-Barraquer et al., 2019; Figure S5D), we also only examined individuals with serologic evidence of prior DENV infection (final n = 14). These individuals were separated into high or low 6-month ZIKV NT_80_ titer groupings based on the tertiles of the 6-month ZIKV NT_80_ titers from the whole DENV-exposed REDS-III cohort (low: n = 6, <230, mid: n = 3, 230–1,240, or high: n = 5, >1,240), which were measured a median of 181 days after index visit (range: 160–196 days; Figure 5A). Of note, there was no significant difference in the age (p = 0.31) or sex distribution (p = 0.53) between the tertiles.

Using a receiver operating characteristic (ROC) analysis, we found that a module 5-skewed score during acute infection was predictive of a high 6-month ZIKV NT_80_ titer, while a module 3-skewed score during acute infection was predictive of a low 6-month titer (area under the curve [AUC] = 0.800; Figure 5B). To investigate which individual features were predictive of high versus low 6-month titers, we returned to an unbiased analysis with the full set of phenotypic features, examining features with significantly different frequencies at the acute time point between the high- and low-titer individuals. While we did not observe an association between the frequencies of antigen-specific populations (e.g., ASCs or HLA-DR+CD38+ CD8+ T cells) at the acute time point and the level of ZIKV neutralizing antibody titers at 6 months post-infection (Figures S5E and S5F), we did find unique sets of features associated with high versus low levels of ZIKV neutralizing antibody titers.

We found that high levels of ZIKV neutralizing antibody titers 6 months post-infection were associated with a significantly higher frequency of 11 cellular features during acute infection (e.g., CD86+ CD14−CD16+ monocytes and pDCs, CD40+ CD14+ monocytes and cDCs, CD69+ NK cells, CD38+ Th1 and Tfh CD4+ T cells, and CD86+, as well as Ki-67+ DN B cells; Figures 5C and 5D). These included multiple activated cell types, eight of which were contained within module 5. Six of the 11 features associated with the high-titer group were specifically expanded in acute infection (indicated as “Changing”). These features also tended to be low in frequency in uninfected individuals (see lighter green colors in “Uninfected [UI] Mean” column). Together, these data suggest that high 6-month ZIKV NT_80_ titers are associated with robust but transient expansion of specific, diverse activated cellular features during the acute phase of infection.

### A cytotoxic immune set point predicts low neutralizing antibody titers after ZIKV infection

In contrast, individuals with low titers of neutralizing ZIKV antibodies 6 months after infection had an acute infection immune signature defined by higher frequencies of cytotoxic T cell features. These included higher granzyme B expression in Tctl CD4+ T cells, a larger TEMRA population in CD8+ T cells, higher Eomesodermin expression in non-naïve CD8+ T cells, a higher overall frequency of non-naïve Vδ2- γδ T cells, and a higher frequency of non-naïve Vδ2- γδ T cells that express granzyme B, T-bet, and Helios (Figures 5C and 5E). Most (8 of 9) of the low-titer-associated features were contained within the stable, cytotoxic-skewed module 3. Unlike the cellular features associated with high 6-month NT_80_ titers, most of the features associated with low 6-month NT_80_ titers were present at high baseline abundance in uninfected individuals (see darker green/blue colors in the Uninfected Mean column), and most (8 of 9) were not dynamically regulated over the course of ZIKV infection. This supported the notion that the cytotoxic-skewed immune signature associated with the development of low neutralizing antibody titers represents a distinct and stable immunologic set point. A higher frequency of cytotoxic-differentiated T cells can relate to a history of infection with other viruses, in particular cytomegalovirus (CMV), and a positive CMV serostatus can be associated with impaired response to vaccination (Bowyer et al., 2020; McElhaney et al., 2012; Merani et al., 2017). However, CMVseropositivity was not significantly associated with the development of low ZIKV NT_80_ titers in our cohort (p = 0.29).

To determine the predictive power of the high- and low-titer-associated features, we again performed an ROC analysis and found that all of the acute infection cellular immune features associated with high or low antibody titers also reliably predicted these two outcomes in this cohort (minimum AUC = 0.833; Figure 5F). Interestingly, several of the low-titer-associated features at the late convalescent time point, after the resolution of infection, remained associated with and were predictive of low 6-month ZIKV NT_80_ titers (Figures 5C, black in the “Late Convalescence” column, and 5G). Collectively, our data suggest that high 6-month ZIKV NT_80_ titers are predicted by an immune state of transiently expanded, highly activated immune cell features during acute infection. In contrast, low 6-month ZIKV NT_80_ titers are instead predicted by a distinct immune set point characterized by a stable, high frequency of cytotoxic-differentiated T cell populations that are not dynamically regulated during acute ZIKV infection.

## DISCUSSION

We present here a deep characterization by mass cytometry of dynamic cellular immune responses to acute ZIKV infection in human adults. Leveraging a well-characterized longitudinal cohort of individuals with viremic ZIKV infection, we found that acute ZIKV infection did not impact the frequency of most major cellular immune populations. However, small populations of highly activated innate and adaptive immune cells were coordinately and transiently expanded during acute infection, and distinct acute-phase immune signatures predicted the persistence of high versus low ZIKV neutralizing antibody titers 6 months after the resolution of infection. Our findings build upon a small but growing set of literature describing cellular immune responses in acute viral infection in humans (Arunachalam et al., 2020; Chandele et al., 2016; Chng et al., 2019; Ellebedy et al., 2016; Kazer et al., 2020; Koutsakos et al., 2021; Liu et al., 2021; Michlmayr et al., 2018; Ndhlovu et al., 2015; Rahil et al., 2020; Rouers et al., 2021; Sekine et al., 2020; Takata et al., 2017; Townsley et al., 2021; Wang et al., 2018), and they suggest immunologic states to target in order to enhance the efficacy of antiviral vaccines.

Our analysis of cellular activation states enabled us to precisely delineate and characterize the coordination between innate and adaptive immune cell populations that respond to acute ZIKV infection. In prior studies, acute ZIKV infection has been associated with activation of some innate immune cell types (Lai et al., 2018) and, in children, an increase in the frequency of monocyte populations that are also a target for viral infection *in vivo* (Michlmayr et al., 2017, 2020). In our study in adults, we observed not only a similar expansion of intermediate CD14+CD16+ monocytes during acute infection but also a transient increase in a suite of activation markers on this cell type in the acute phase. Additionally, we identified a cluster of CD14+ monocytes that were present at a higher frequency during acute infection (c49) with distinct co-regulated markers denoting proliferation, activation, or differentiation states. Among adaptive immune cells, HLA-DR+CD38+ non-naïve CD8+ T cells were expanded at the acute time point, consistent with other acute viral infections (Chandele et al., 2016; Koutsakos et al., 2021; Wang et al., 2018). We found that these cells were contained in three distinct clusters of cells that co-express different combinations of activation markers. Acute ZIKV infection was also associated with activation of T helper type 1 (Th1) and Tctl T cell CD4+ T cell subsets. Finally, using gating strategies to identify populations of B cells enriched for antigen-specific cells in other infections (Andrews et al., 2019; Ellebedy et al., 2016), we identified an expansion of Tbet+ASCs during acute ZIKV infection.

Our study describes the diverse and coordinated activation of cellular immune responses during acute ZIKV infection in human adults. Compared with the baseline correlations that exist in the uninfected state, we found that acute ZIKV infection drove new coordination between different immune cell types and across the innate and adaptive immune system. The correlations unique to acute ZIKV infection (e.g., positive correlations in the proportion of CD38+ pDCs and CD38+ Th1 CD4+ T cells, or between CD40+ cDCs and Ki-67+ DN B cells) may reflect interactions that are essential to mount a productive antiviral immune response. Further exploration of the correlated features in acute infection revealed two distinct modules that were inversely correlated: one (module 5) contained features reflecting transiently elevated activated cell populations, while the second (module 3) contained features reflecting stable/unchanging cytotoxic cell populations. Remarkably, these two acute infection immune signatures, which we identified using an unbiased analysis approach, appear to truly reflect distinct immune states that differentially impact and predict the development and maintenance of high neutralizing antibody responses.

High titers of ZIKV neutralizing antibodies are likely critical for protective immunity in humans, and they are a key target for ZIKV vaccines (Diamond et al., 2019). Six months following infection, participants across our cohort had a greater than 100-fold difference in ZIKV neutralizing antibody titers. Other than a positive association with prior DENV serostatus observed here and in other studies (Rodriguez-Barraquer et al., 2019), little is known about what parameters predispose some individuals to maintain higher versus lower ZIKV neutralizing antibody titers. Interest-ngly, as has been in observed in severe acute respiratory syndrome coronavirus 2 (SARS-CoV-2) infection (Koutsakos et al., 2021), the frequency of ASCs during acute ZIKV infection did not correlate with antibody levels in convalescence. We did, however, find several other acute-phase cellular features that were associated with and predictive of high versus low neutralizing antibody titers, many of which have plausible roles in augmenting a productive B cell response. For example, CD86 expression on pDCs and monocytes and CD40 expression on cDCs and monocytes can mediate enhanced antigen presentation to and priming of helper CD4+ T cells, interferon gamma (IFN-γ) produced by activated Th1 cells or NK cells can promote B cell activation, and activated Tfh CD4+ T cells can provide direct help to differentiating B cells. Further investigation could elucidate whether robust induction of these same activated cell populations also predicts the long-term immunogenicity of vaccines for ZIKV and other viral infections.

In contrast to the dynamically regulated acute-phase cellular immune features associated with high ZIKV neutralizing antibody titers, a higher frequency of T cells with cytotoxic-differentiation features were associated with low 6-month ZIKV neutralizing antibody titers and were predictive of levels of 6-month ZIKV neutralizing antibody titers. Most of these features did not dynamically change over the course of infection and were themselves inversely correlated with the cellular immune features associated with high 6-month ZIKV neutralizing antibody titers (similar to the inverse correlation between modules 3 and 5 in the correlation matrix). Several of the low-titer-associated cytotoxic features were also present at higher levels in the low-titer individuals 3–6 months after resolution of the infection, suggesting that they may represent a stable biological state that is likely reflective of their history of prior antigen encounters. This state is distinct from the small populations of virus-specific (e.g., HLA-DR+CD38+) T cells that are transiently expanded in acute infection (Chandele et al., 2016; Koutsakos et al., 2021; Wang et al., 2018). The stability of these features suggests that a cytotoxic immune set point may identify individuals predisposed to have a blunted activation response to acute infection that then leads to impaired neutralizing antibody responses. In general, a more cytotoxic-skewed T cell compartment can be a sign of immune senescence, which can in turn be associated with a reduced capacity to generate functional antigen-specific responses after vaccination (Bowyer et al., 2020; McElhaney et al., 2012). Thus, in addition to identifying candidate biomarkers of a responsive immune signature that may be useful for predicting the formation of a robust neutralizing antibody response to other infections or vaccination, our study also provides insight into potential markers of an immune state that impairs the formation of protective immunity after acute viral infection. Future studies should explore the generalizability of our findings to other infections and vaccination and the underlying causes of these distinct immune signatures.

Our study providesa first in-depth characterization of the cellular immune response to acute ZIKV infection in human adults and relates distinct acute-phase cellular immune signatures to the development of high or low titers of durable neutralizing antibodies. Our approach offers a powerful tool to test whether these features also predict immunogenicity of vaccines for ZIKV and other viral infections, such as SARS-CoV-2, for which neutralizing antibodies play a major role in protection. Our findings suggest that targeted therapeutic approaches in individuals predicted to have poor neutralizing antibody responses to vaccination (e.g., different adjuvants or a higher dose of vaccine) might increase acute-phase immune activation and subsequently promote enhanced long-term protective antiviral immunity.

### Limitations of the study

Our study has some important limitations. Although we have made an effort to control for the variance introduced by sampling time, it was not possible to align participants according to the exact date they were infected. Our study included only otherwise healthy individuals who presented for volunteer blood donation and does not include pregnant individuals or infants, who are key populations affected by this infection. Finally, while it is likely that neutralizing antibodies play a key role in immunologic protection from ZIKV (Khoury et al., 2021), a titer that correlates with protection in humans has not yet been identified (Richner and Diamond, 2018), and other antibody functions (Maciejewski et al., 2020) and/or other types of immune responses (Abbink et al., 2016; Hassert et al., 2019; Larocca et al., 2016) may also be critical for robust protection.

## STAR★METHODS

### RESOURCE AVAILABILITY

#### Lead contact

Further information and requests for resources and reagents should be directed to and will be fulfilled by the lead contact, Rachel Rutishauser (rachel.rutishauser@ucsf.edu).

#### Materials availability

This study did not generate new unique reagents.

#### Data and code availability

- De-identified raw fcs files with mass cytometry data were deposited on Mendeley: https://doi.org/10.17632/5cn6cy97b7.2. All data generated from manual gating and clustering analysis are provided in supplemental tables.
- This paper does not report original code.
- Any additional information required to reanalyze the data reported in this paper is available from the lead contact upon request.

### EXPERIMENTAL MODEL AND SUBJECT DETAILS

We characterized the cellular immune response to acute and resolving ZIKV infection in the peripheral blood of 25 otherwise healthy adults (negative for HIV, hepatitis B and C infections) who had viremic ZIKV infection between 2015 and 2016 at the time of blood donation in Puerto Rico (“index” visit). Participants were selected from the prospective REDS-III cohort (see Table S1 for demographic and clinical characteristics) (Musso et al., 2017; Stone et al., 2020; Williamson et al., 2020). Peripheral blood mononuclear cells (PBMCs) from these ZIKV+ individuals were sampled longitudinally at up to three intervals after the index visit: (1) within 5–12 days after the index visit (“acute”; median 8 days), (2) within 15–27 days after index visit (“early convalescence”; median 21 days), and (3) within 85–189 days after index visit (“late convalescence”; median 91 days; see Figure 1A for sampling schema). 19 of 25 individuals had longitudinal sampling of PBMCs available at all three timepoints. ZIKV viral load and antibody measurements were performed at these and additional timepoints (see Figure 1B). Participants were asked about the presence of 6 symptoms at each visit (fever, rash, joint or bone pain, body or muscle pain, painful or red eyes, headache). At the first (“acute”) PBMC collection time point, 40% of the ZIKV+ donors had ≥ 3 symptoms present. Two groups of ZIKV-uninfected (“ZIKV−”) individuals were also included in our analyses. The ZIKV− participants were recruited from a larger cohort of blood donors based at the same blood donation clinics as the ZIKV+ participants (with the same sample processing protocols), with samples collected in the years prior to the onset of the ZIKV epidemic in Puerto Rico. Samples from the first ZIKV− cohort (single timepoint, n = 8) were processed and run on the mass cytometer in parallel with the samples from the ZIKV+ individuals. Samples from the longitudinal ZIKV− cohort (n = 6 participants across three timepoints) were run separately. All samples were obtained with appropriate informed consent and ethics committee approval of the University of California San Francisco.

### METHOD DETAILS

#### Viral load and antibody measurements

ZIKV viral load, antibody levels, and ZIKV and DENV neutralizing antibody measurements were performed as described previously (Stone et al., 2020; Williamson et al., 2020). In brief, ZIKV viral load was measured by quantitative PCR. Anti-Zika virus IgM and IgG were measured by antibody-capture ELISA using recombinant ZIKV antigen kindly provided by the US Centers for Disease Control and Prevention (CDC) and as previously described (Lanciotti et al., 2008; Williamson et al., 2017). ZIKV neutralizing titers were measured using a ZIKV reporter viral particle neutralization titration assay (Integral Molecular, Philadelphia, PA) (Whitbeck et al., 2020), and index donations were tested for pre-existing DENV IgG with the Detect IgG ELISA (InBios; Seattle, WA).

#### PBMC preparation and mass cytometry staining

Whole peripheral blood was collected at the clinical sites, shipped overnight at ambient temperature to Vitalant, San Francisco, CA, USA, where they were processed and cryopreserved within 24 h of collection and then stored in liquid nitrogen as previously described (Musso et al., 2017). Mass cytometry experiments were performed over the course of five separate experiments, with normalization between experiments performed as outlined below. PBMCs were thawed, and only samples with >70% viability were used for analysis (most were >90% viable after thawing by the Muse Cell Analyzer [Millipore Sigma, Burlington, MA, USA]) (Odorizzi et al., 2018; Owen et al., 2007). We stained 2–4 million cells per panel in two mass cytometry panels (see STAR Methods for antibody clones and metals), following a previously published protocol (Spitzer et al., 2017) with the following modifications. Briefly, we marked dead cells by incubating the samples for one minute with 25 mM Cisplatin (Sigma-Aldrich, St. Louis, MO, USA) in phosphate buffered saline (PBS) plus EDTA, performed surface staining with metal-tagged antibodies in PBS with 0.5% bovine serum albumin (BSA) for 30 min at room temperature, fixed and permeabilized cells following manufacturer’s instructions for the eBioscience Foxp3/Transcription Factor Staining Buffer Set (Thermo Fisher Scientific, Waltham, MA, USA), barcoded samples using mass-tag cellular barcoding reagents diluted in Maxpar Barcode Perm Buffer (Fluidigm, South San Francisco, CA, USA) as described previously (Spitzer et al., 2017), combined up to twenty barcoded samples into a single tube, performed intracellular staining with antibodies diluted in eBioscience Foxp3/Transcription Factor kit perm wash (Thermo Fisher Scientific), fixed cells in freshly prepared 2% paraformaldehyde (Electron Microscopy Sciences, Hatfield, PA, USA) in the presence of a DNA intercalator (Ornatsky et al., 2008), and then washed and ran cells on the Fluidigm CyTOF 2 mass Cytometer within one week of staining.

#### Mass cytometry data processing

##### Data quality control

Following data acquisition, the FCS files were normalized across experiments using bead standards and the data normalization algorithm using the R package ‘premessa.’ The live cell events were debarcoded using a single-cell debarcoding algorithm (Zunder et al., 2015) and we analyzed >25,000 (mostly >50,000) cells per sample. From the individual sample files, normalization beads were excluded based on Ce140 and Eu153 signal, single cell events were identified based on Ir191 DNA signal measured against event length, and CD45− or Pt195+ dead cells were excluded. Potential batch effects were minimized by including samples from the same individual in the same experiment. Spillover between the Yb173 and Yb174 channels was compensated based on the CyTOF metal purity matrix (Han et al., 2018) using flowcore (Ellis et al., 2019). Gating was performed using CellEngine (CellCarta, Montreal, Canada).

##### Manual gating

Traditional hierarchical gating was applied to identify 12 “landmark” immune populations: CD14+ “classical” monocytes, CD14− CD16+ “non-classical” monocytes, classical and plasmacytoid dendritic cells [cDC and pDC, respectively], basophils, CD56^bright^ and CD56^dim^ natural killer cells, regulatory CD4+ T cells, non-regulatory CD4+ T cells, CD8+ T cells, γδ T cells as stained by either a pan-γδ T cell receptor (TCR) antibody or an antibody that only recognizes γδ T cells with the Vδ2 chain (see Figure S1 for gating strategy) as well as well-defined adaptive immune subsets (see Figure S2 for the identity of these populations). Within each of the “parent” cell types, we manually gated positive and negative populations of biologically relevant phenotypic markers from the two mass cytometry panels (see Table S2 for markers assessed on each “parent” population). For each of the parent cell types, we only included phenotypic markers for which we could clearly gate a positive population above background antibody staining levels.

##### Clustering by statistical SCAFFoLD

We generated SCAFFoLD maps using the Scaffold R package. As described previously (Spitzer et al., 2015, 2017), using all of the live CD45+ leukocytes collected across participants and timepoints for each staining panel, we applied an unsupervised clustering algorithm based on the CLARA clustering algorithm to partition cells into a user-defined number of clusters (100 clusters per staining panel). We excluded Ki-67 and Granzyme B to avoid having functional markers cluster cells across cell types together. Landmark populations were gated as outlined in Figure S1 (for cluster analysis, NK cells were treated as one population). We next generated force-directed graphs (SCAFFoLD maps) to visualize the association of each cluster with its likely parent landmark population. We excluded from our downstream analysis clusters that contained <20 cells in >80% of samples (12 clusters in Panel 1, 2 clusters in Panel 2) as well as clusters that contained cells that did not have the expected expression of classical landmark population (e.g., we excluded a cluster of cells that clustered with the CD8+ T cells but appeared to co-express the B cell marker, CD19 and may potentially represent doublets [median 0.09% of total CD8+ T cells at the acute timepoint]; all together, these 9 clusters in Panel 1 and 7 clusters in Panel 2 comprised 0.08% and 0.13% of the total live population at the acute timepoint). Cell clusters were thus determined to be “reliably” assigned to landmark cell populations if they were not excluded based on these criteria and if they were identified in Panel 1 for innate immune cells (total 17 classical and 3 non-classical monocyte, 9 NK cell, 4 cDC, 1 pDC clusters) and B cells (total 14 clusters) and Panel 2 for T cell phenotypes (total 6 CD4+ Treg, 20 non-Treg CD4+, 14 CD8+, and 2 non-Vδ2 γδ T cell clusters). In the SCAFFoLD maps depicted, a representative map from one participant at timepoint 1 is shown.

### QUANTIFICATION AND STATISTICAL ANALYSIS

#### Change in manually gated population and cell cluster frequencies over time

To measure the change in abundance of manually gated cell features (e.g., landmark and sub-landmark populations and populations expressing individual phenotypic markers) and cell clusters, the frequency of each feature (expressed as a % of the parent population) was log transformed with a constant factor of 1/10E6 or 1/10E3, respectively. Log-transformed values were adjusted for participant age and sex using a linear regression and the residuals (log-adjusted abundance) were used in downstream analyses. For age and sex, the median (± standard deviation) contribution of each of these factors to the variance for individual features was 1.63 (±5.83)% and 1.17 (±3.16)%, respectively. Age/sex contribution to variance for individual features can be found in Data S2. The change over time for the log-adjusted feature abundance between the “acute,” “early convalescent” and “late convalescent” visits was assessed using a linear mixed effect (LME) model with the nlme R package (Pinheiro et al., 2019) with log-transformed days since index visit as a fixed effect and participant ID as a random effect. The p values for each group of features were adjusted for multiple testing correction by Benjamini Hochberg with an FDR cutoff of 5% for a significant effect of time since index visit on feature abundance. For 95% confidence interval graphs, line graphs were generated in R using the package ggplot2 (Wickham, 2016). The 95% confidence intervals for the median values were calculated by bootstrapping with 1000 iterations.

#### PCA analysis

The log-adjusted manually gated features that were present across all ZIKV infected and cross-sectional uninfected samples (281 of 324 total features in the dataset) were used for principal component analysis with the function PCA (parameters: "scale.unit = TRUE", ncp = 5) from the R package FactoMineR (Lê et al., 2008). The samples were visualized in PCA space with PC1 and PC2 values as the coordinates using factoextra and ggplot2 in R.

#### Heatmaps

Heatmaps were made in R using the package ComplexHeatmap (Gu et al., 2016). For the manually gated features and cluster features summary heatmaps, the row/column orders, respectively, were determined using the R package seriation (Hahsler et al., 2008) with the travelling salesperson problem (TSP) method.

#### Network correlation analysis

Pairwise Spearman correlations were calculated on the log-adjusted feature abundances from samples at the acute visit for participants (n = 17) who were previously exposed to Dengue and in an early stage of infection (pre-IgM at the time of Index visit). The p values were adjusted with the Benjamini-Hochberg method. The correlation matrix was hierarchically clustered using complete linkage based on Euclidean distance to create correlation modules. For the relationship between modules, the average value was calculated across all significant correlations (p_adj<0.05) between features within each module. For the module 5 - module 3 score, each module score is the sum of the z-score scaled log-adjusted cellular features within the module. The 95% confidence intervals for the correlation in the infected samples for each pairwise feature comparison was calculated using bootstrapping with 1000 iterations. For each significant correlation (p adjusted<0.05), the correlation was categorized as “shared” with the uninfected cohort if the correlation value in the uninfected cohort fell within the 95% confidence interval from the infected samples or had the same sign as the infected correlation and a magnitude greater than the 95% confidence interval magnitude maximum. Otherwise, the correlation was categorized as “unique.” Fisher’s exact test was used to determine odds ratio for correlations being unique to ZIKV as the exposure (versus being “shared” with the uninfected) and the indicated correlation attribute as the outcome. The circular network graph was visualized using ggplot2 and the marker network graph was visualized with igraph (Csardi, 2006).

#### Antibody associations

The NT_80_ titers at the 6-month timepoint of the DENV-exposed, ZIKV+ individuals from the larger REDS III cohort were classified into antibody tertiles. The association between age and sex and the 6-month NT_80_ titer groupings was assessed on the entire REDS-III cohort using one-way ANOVA and a Chi-square test of independence, respectively. To test the association between cellular immune phenotypes and ZIKV neutralizing antibody titers, we used acute or late-convalescent visit samples from participants who had not yet formed IgM at the index visit, were DENV-exposed, and who had 6-month NT_80_ titers available (n = 14). Exact permutation tests were used to test for significant differences in the log-adjusted cellular features (age- and sex-adjusted) between samples from participants in the high versus low tertiles (n = 5 in high group and n = 6 in low group). The association between CMV IgG seropositivity and 6-month NT_80_ titers was assessed using the Wilcoxon Rank Sum test based on a larger subset of REDS-III study participants for whom CMV serostatus were available (n = 10 CMV+, n = 23 CMV−).

#### Antibody associations predictive modelling

We used pROC (Robin et al., 2011) to plot ROC curves with log-adjusted feature abundance at the acute or late convalescent visit as the predictor and 6-month NT_80_ antibody titer category (e.g., “High” or “Low”) as the response for each participant. The 95% CI for the AUC values were computed with the default “DeLong” method.

## Supplementary Material

1

2

3

4

## Figures and Tables

**Table:** KEY RESOURCES TABLE

REAGENT or RESOURCE	SOURCE	IDENTIFIER
Antibodies		
Mass cytometry antibodies: metal-antigen (clone)	Self-conjugated unless from Fluidigm	
Y89-CD45 (clone HI30)	Fluidigm	Cat#3089003B; RRID:AB_2661851
In113-CD14 (clone M5E2)	BioLegend	Cat#301802; RRID:AB_314184
In115-CD123 (clone 6H6)	BioLegend	Cat#306002; RRID:AB_2661822
La139-CD33 (clone WM53)	BioLegend	Cat#303402; RRID:AB_314346
Ce140-CD38 (clone HIT2)	BioLegend	Cat#303502; RRID:AB_314354
Pr141-CD3 (clone UCHT1)	BioLegend	Cat#300402; RRID:AB_2661835
Nd142-CD19 (clone H1B19)	BioLegend	Cat#302202; RRID:AB_2661817
Nd143-CXCR3 (clone G025H7)	BioLegend	Cat#353702; RRID:AB_10983073
Nd144-CD11b (clone ICRF44)	BioLegend	Cat#301302; RRID:AB_314154
Nd145-CD4 (clone RPA-T4)	BioLegend	Cat#300502; RRID:AB_314069
Nd146-CD8 (clone RPA-T8)	BioLegend	Cat#301002; RRID:AB_2661818
Sm147-CD11c (clone Bu15)	BioLegend	Cat#337202; RRID:AB_1236381
Nd148-CD16 (clone 3G8)	BioLegend	Cat#302001; RRID:AB_314201
Sm149-CD138 (clone DL-101)	BioLegend	Cat#352302; RRID:AB_10915555
Eu151-CD21 (clone Bu32)	BioLegend	Cat#313502; RRID:AB_416326
Sm152-gdTCR (clone 11F2)	Fluidigm	Cat#3152008B; RRID:AB_2687643
Eu153-CD45RA (clone HI100)	BioLegend	Cat#304102; RRID:AB_314406
Sm154-CD40 (clone 5C3)	BioLegend	Cat#334302; RRID:AB_1236384
Gd156-PDL1 (clone 29E.2A3)	BioLegend	Cat#329702; RRID:AB_940372
Gd157-CD69 (clone FN50)	BioLegend	Cat#310902; RRID:AB_314837
Gd158-CD27 (clone O323)	BioLegend	Cat#302802; RRID:AB_2661825
Gd160-Tbet (clone 4B10)	BioLegend	Cat#644802; RRID:AB_1595503
Dy161-CTLA4 (clone 14D3)	Fluidigm	Cat#3161004B; RRID:AB_2687649
Dy162-CD80 (clone 2D10.4)	Fluidigm	Cat#3162010B; RRID:AB_2811101
Dy163-CD86 (clone IT2.2)	BioLegend	Cat#305401; RRID:AB_314521
Ho165-CD24 (clone MI5)	BioLegend	Cat#311102; RRID:AB_314851
Er166-NKG2D (clone ON72)	Fluidigm	Cat#3166016B; RRID:AB_2892110
Er167-FCRL5 (clone 509f6)	BioLegend	Cat#340302; RRID:AB_2104586
Er168-Ki67 (clone B56)	Fluidigm	Cat#3168007B; RRID:AB_2800467
Tm169-CD71 (clone CY1G4)	BioLegend	Cat#334102; RRID:AB_1134247
Er170-IgD (clone IA6-2)	BioLegend	Cat#348202; RRID:AB_10550095
Yb171-CD20 (clone 2H7)	BioLegend	Cat#302302; RRID:AB_314250
Yb172-BDCA1 (clone L161)	BioLegend	Cat#331502; RRID:AB_2661820
Yb173-IgM (clone MHM-88)	BioLegend	Cat#314502; RRID:AB_493003
Yb174-HLA-DR (clone L243)	BioLegend	Cat#307602; RRID:AB_314680
Lu175-PD-1 (clone EH12.2H7)	BioLegend	Cat#329902; RRID:AB_940488
Yb176-CD56 (clone HCD56)	Fluidigm	Cat#3176008B; RRID:AB_2661813
Sm149-CCR4 (clone 205410)	R&D	Cat#MAB1567; RRID:AB_2074395
Nd150-OX40 (clone A019D5)	BioLegend	Cat#351302; RRID:AB_10718513
Eu151-ICOS (clone C398.4A)	BioLegend	Cat#313539; RRID:AB_2810475
Sm154-CX3CR1 (clone 2A9-1)	BioLegend	Cat#341602; RRID:AB_1595422
Gd155-CCR6 (clone G034E3)	BioLegend	Cat#353402; RRID:AB_10918625
Tb159-Vd2 (clone B6)	BioLegend	Cat#331402; RRID:AB_1089226
Dy162-FOXP3 (clone PCH101)	BioLegend	Cat#3162011a; RRID:AB_2687650
Dy164-EOMES (clone WD1928)	ThermoFisher	Cat#14-4877-82; RRID:AB_2572882
Ho165-CD127 (clone A019D5)	BioLegend	Cat#351302; RRID:AB_10718513
Er166-TIGIT (clone A15153G)	BioLegend	Cat#372702; RRID:AB_2632714
Er167-CCR7 (clone G043H7)	BioLegend	Cat#353202; RRID:AB_10945157
Tm169-CD25 (clone 2A3)	Fluidigm	Cat#3169003B; RRID:AB_2661806
Yb171-CXCR5 (clone RF8B2)	Fluidigm	Cat#3171014B; RRID:AB_2858239
Yb172-Helios (clone 22F6)	BioLegend	Cat#137202; RRID:AB_10900638
Yb173-Granzyme B (clone GB11)	BioRad	Cat#MCA2120; RRID:AB_2114582
Biological samples		
Cryopreserved human PBMCs and plasma	REDS-III study participants	Demographic Data available in Table S1
Chemicals, peptides, and recombinant proteins		
Cisplatin	Sigma-Aldrich	Cat #P4394
eBioscience FoxP3/Transcription Factor Staining Buffer Set	Thermo Fisher Scientific	Cat #00-5523-00
Maxpar Barcode Perm Buffer	Fulidigm	Cat #201057
Paraformaldehyde	Electron Microscopy Sciences	Cat #15710
Intercalator	Fluidigm	Cat #201103A
Deposited data		
Mass cytometry data	This paper	https://doi.org/10.17632/5cn6cy97b7.2
Software and algorithms		
CellEngine	CellCarta	https://cellcarta.com/cellenginesoftware/
R 3.6.1	The R Foundation	https://www.r-project.org/
premessa 0.1.8	R package	https://github.com/ParkerICI/premessa
flowCore 1.50.0	Ellis et al., 2019	RRID:SCR_002205
ggplot2 3.2.1	Wickham, 2016	RRID:SCR_014601
nlme 3.1-140	Pinheiro et al., 2019	RRID:SCR_015655
factoextra 1.0.5	Kassambara and Mundt, 2017	RRID:SCR_016692
FactoMineR 1.42	Lê et al., 2008	RRID:SCR_014602
seriation 1.2.8	Hahsler et al., 2008	https://cran.r-project.org/package=seriation
ComplexHeatmap 2.1.1	Gu et al., 2016	RRID:SCR_017270
SCAFFoLD	Spitzer et al., 2015	https://github.com/SpitzerLab/statisticalScaffold
igraph 1.2.4.1	Csardi, 2006	RRID:SCR_019225
pROC 1.17.0.1	Robin et al. 2011	https://CRAN.R-project.org/package=pROC

## Highlights

- We investigate coordinated immune activation during acute ZIKV infection
- Individuals have distinct cellular immune signatures during acute ZIKV infection
- High immune activation in acute ZIKV predicts high neutralizing antibody levels
- A stable cytotoxic-skewed set point predicts low neutralizing antibody levels
