# Supplementary material for: A cytotoxic-skewed immune set point predicts low neutralizing antibody levels after Zika virus infection
Source: Cell Rep. Author manuscript; Available in PMC 2022 May 31. (PMC9151348; doi:10.1016/j.celrep.2022.110815)
Supplement: 1 [file NIHMS1808942-supplement-1.pdf]

Cell Reports, Volume *39*

Supplemental information

A cytotoxic-skewed immune set point predicts low neutralizing antibody levels after Zika virus infection

Elizabeth E. McCarthy, Pamela M. Odorizzi, Emma Lutz, Carolyn P. Smullin, Iliana Tenvooren, Mars Stone, Graham Simmons, Peter W. Hunt, Margaret E. Feeney, Philip J. Norris, Michael P. Busch, Matthew H. Spitzer, and Rachel L. Rutishauser

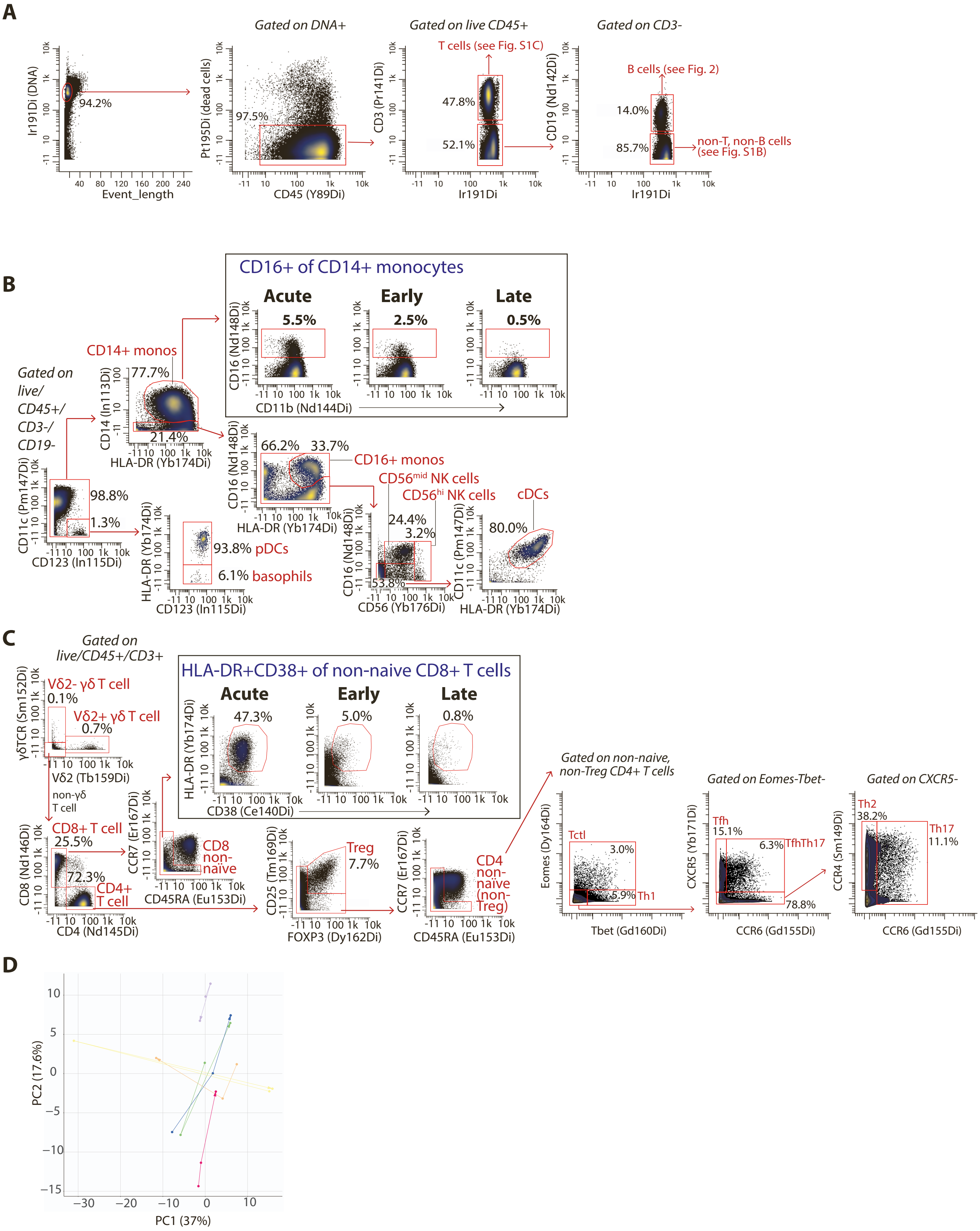

Figure S1. CyTOF gating strategy (related to: STAR methods) and Principal Component Analysis (PCA) of uninfected participants, (related to: Fig. 1C). Gating strategy for (A) landmark populations, (B) innate immune cells, and (C) T cells. (D) PCA representation of all manually gated parameters measued on PBMCs from ZIKV-uninfected control participants (N=6) at longitudinal timepoints.

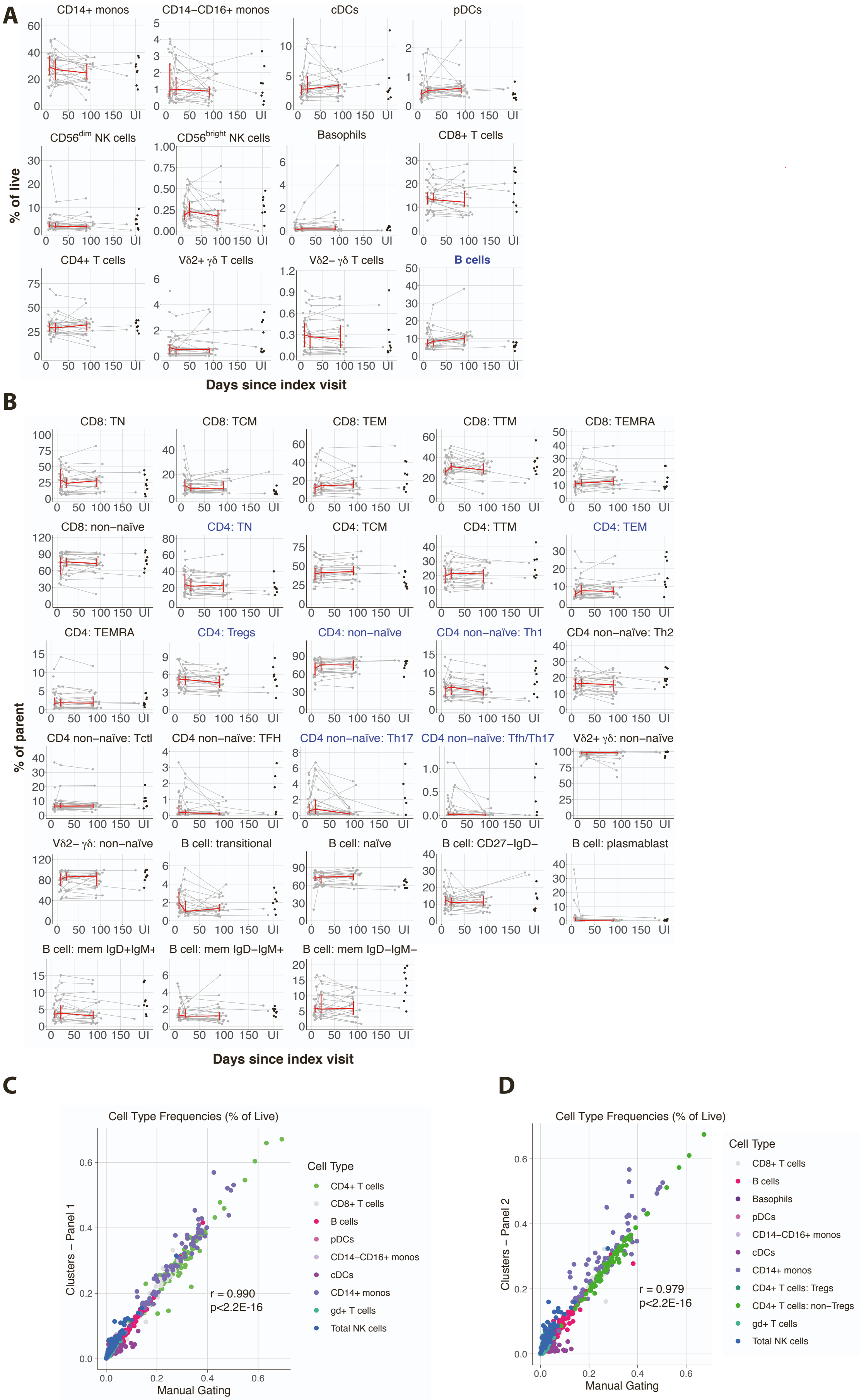

Figure S2. Landmark and sub-landmark population abundance in acute and convalescent ZIKV infection (related to: Figs. 1E-G). Line plots of frequency of indicated (A) landmark cell type and (B) adaptive immune subset for each participant versus time since index visit. Red line connects median values at each sampling timepoint with error bars for +/- 95% CI. Scatterplot for feature abundance from cross-sectional uninfected (UI) cohort shown on the far right. Features with p_adj< 0.05 have blue colored titles. p_adj values obtained by LME model fit with Benjamini-Hochberg FDR correction. High concordance in landmark cell population frequencies as measured by manual gating versus SCAFFoLD clustering analysis in Panel 1 (C) and Panel 2 (D). N=25 ZIKV+ and N=8 ZIKV- participants.

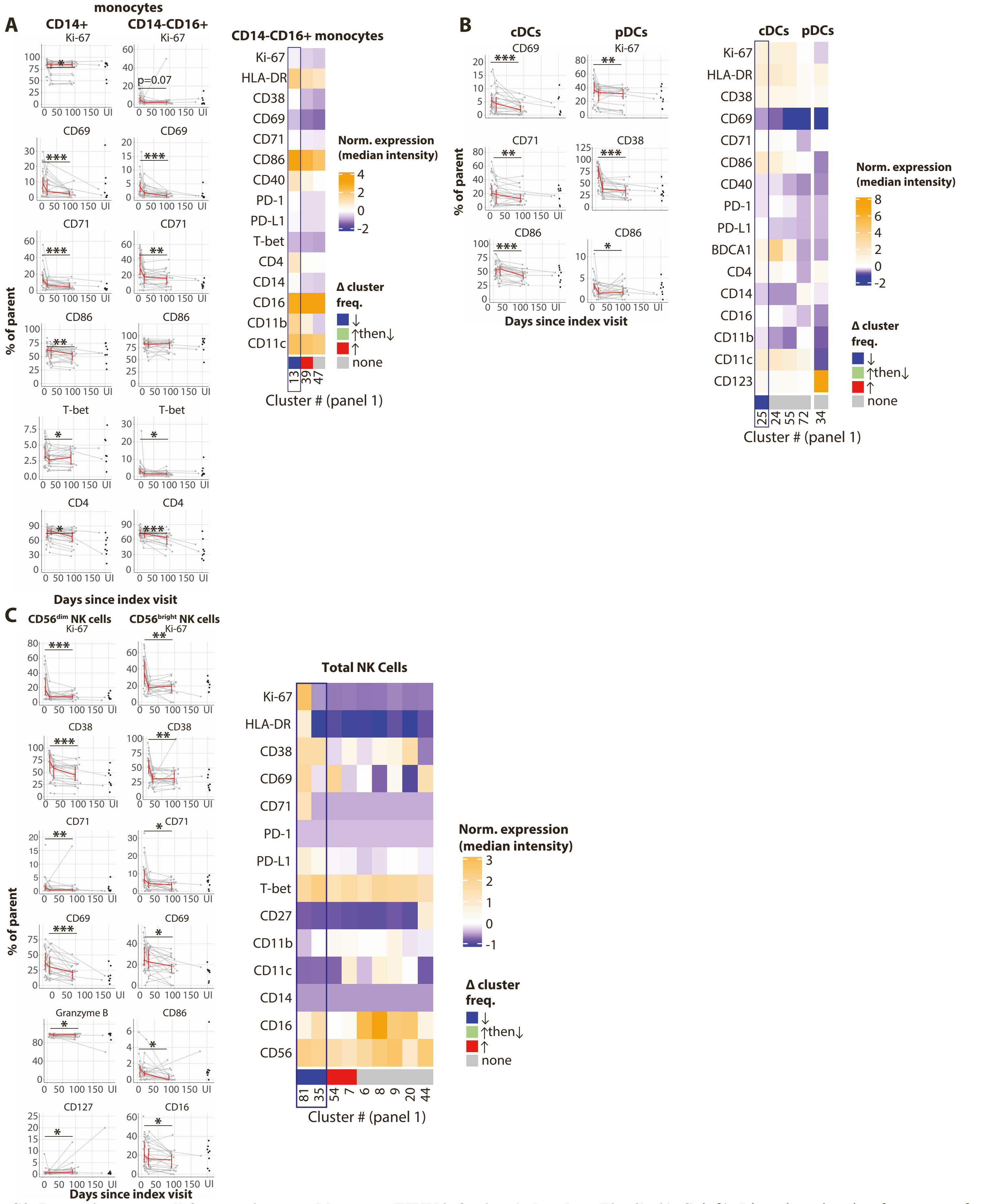

Figure S3. Innate immune cell features impacted by acute ZIKV infection (related to: Fig. 2). (A-C, left): Line plots showing frequency of phenotypic features (% of cells that express each marker) versus time since index visit (N=25). Red line connects median values at each sampling timepoint with error bars for +/- 95% CI. Feature abundance from cross-sectional uninfected (UI) cohort (N=8) shown on the far right. *p_adj<0.05, **p_adj<0.01, ***p_adj<0.001 (p_adj values obtained by LME model fit with Benjamini-Hochberg FDR correction). (A-C, right): Heatmaps showing z-score normalized median expression of indicated markers (rows) for each landmark cell population-associated cell cluster (column). Column annotation indicates clusters that significantly decrease (blue), increase (red), increase and then decrease (green), or remain unchanged (grey) in abundance (as a % of the parent population; p_adj<0.05). N=25 ZIKV+ and N=8 ZIKV- participants.

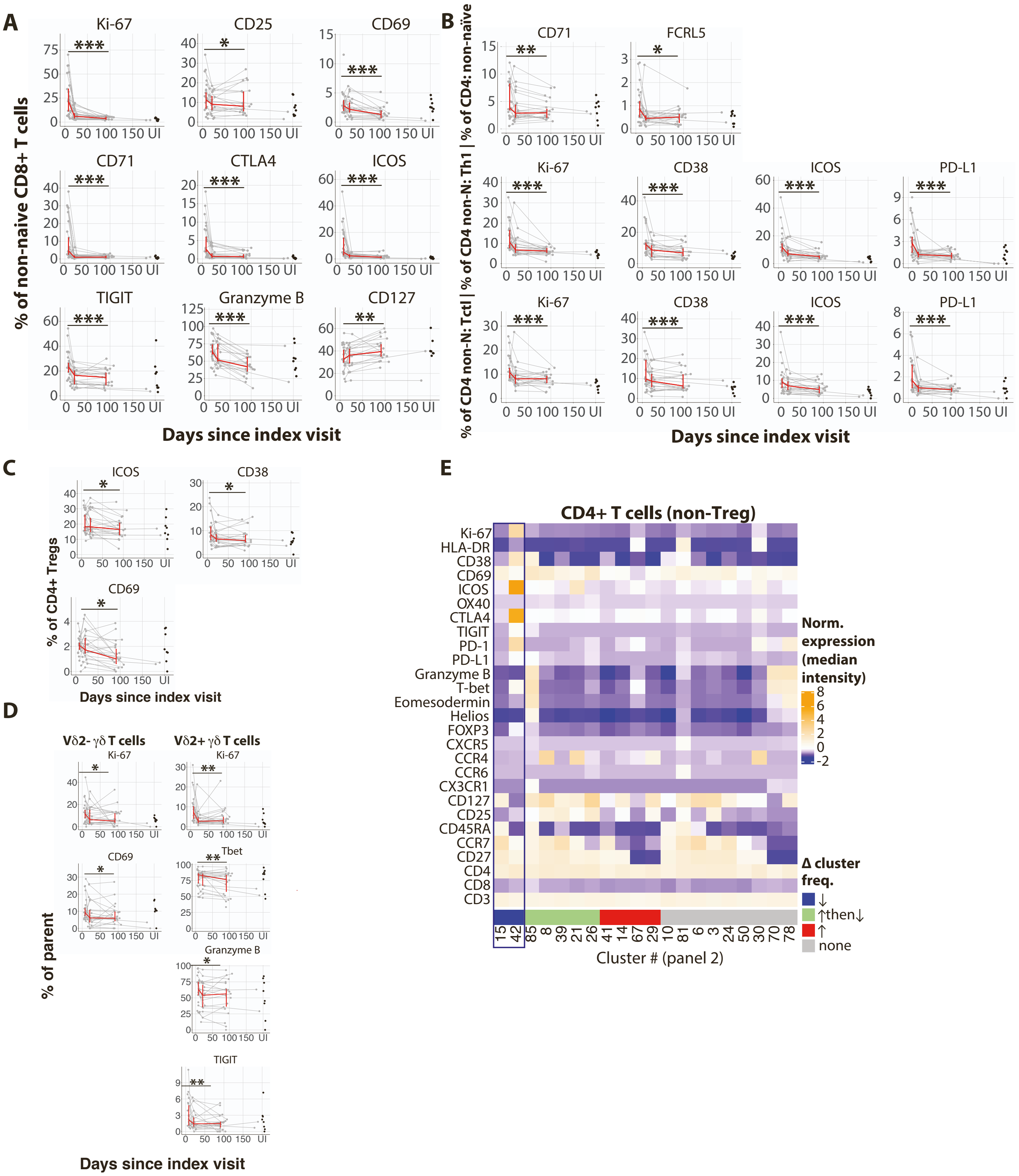

Figure S4. T cell features impacted by acute ZIKV infection (related to: Fig. 2).
Significantly changing features (shown as % of parent cells that express each marker) for (A) non-naive CD8+ T cells, (B) non-Treg non-naive CD4+ T cells, (C) CD4+ Tregs, and (D) γδ T cells for each participant versus time since index visit. Red line connects median values at each sampling timepoint with error bars for +/- 95% CI. Scatterplot for feature abundance from cross-sectional uninfected (UI) cohort shown on the far right. *$p_{adj}<0.05$, **$p_{adj}<0.01$, ***$p_{adj}<0.001$ (p_{adj} values obtained by LME model fit with Benjamini-Hochberg FDR correction). (E) Phenotype (z-scored median expression of each marker) of non-Treg non-naive CD4+ T cell clusters that significantly decrease (blue), increase (red), increase and then decrease (green) or remain unchanged in abundance ($p_{adj}<0.05$). N=25 ZIKV+ and N=8 ZIKV- participants.

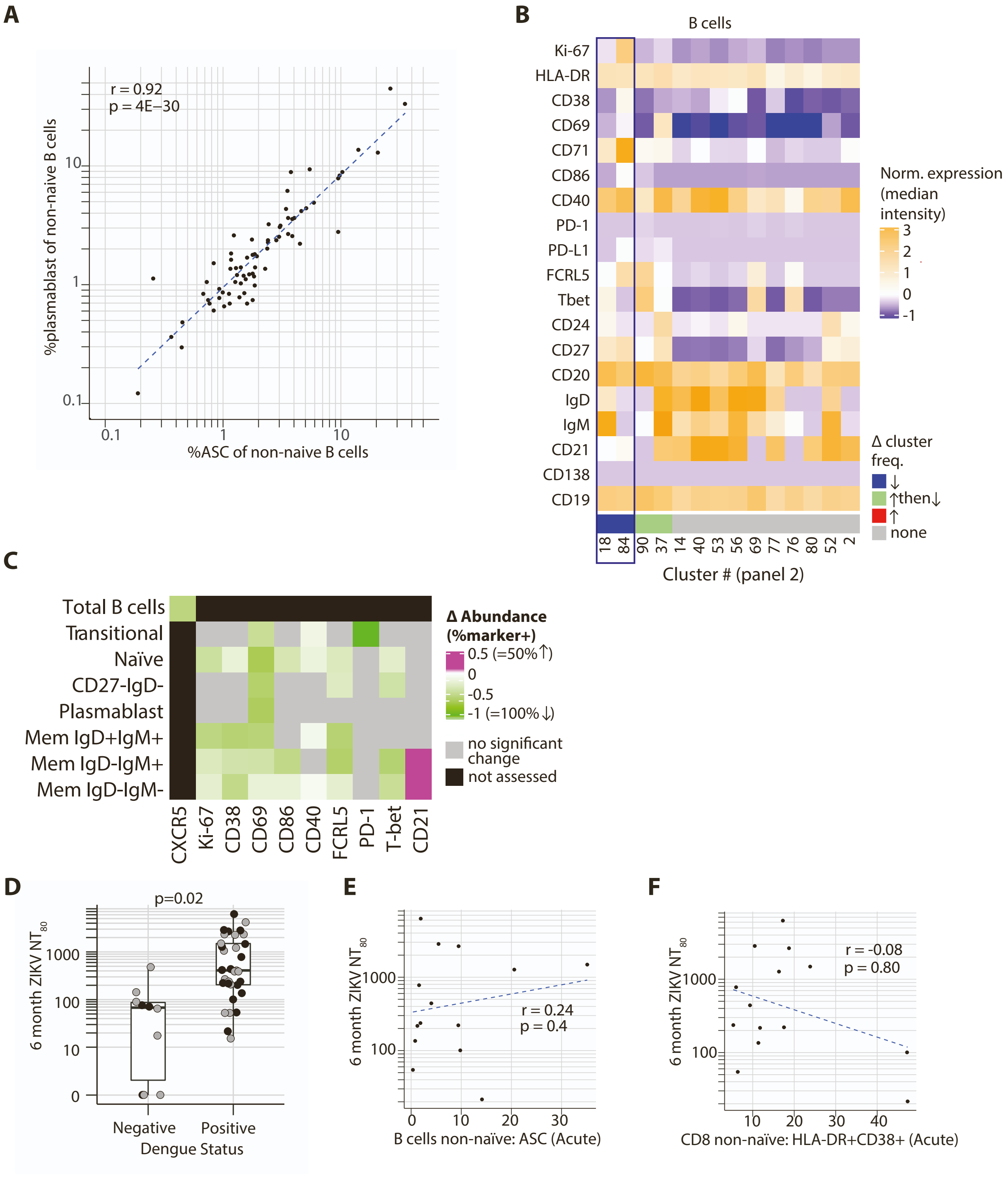

Figure S5. B cell dynamics in ZIKV infection (related to: Fig. 2) and characteristics associated with ZIKV neutralizing antibody titers 6 months after infection (related to: Fig. 5).
(A) Correlation plot between the frequency of plasmablast and ASC B cell populations (as a % of non-naïve B cells; Spearman's r with regression line). (B) Phenotype (z-scored median expression of each marker) of B cell clusters that significantly decrease (blue), increase (red), increase and then decrease (green), or remain unchanged (grey) in abundance (as a % of the total B cell population; p_adj<0.05). (C) Relative change in the frequency [(late convalescent - acute)/acute] of the expression of individual activation markers on B cell subsets (median shown). Colors indicate markers with a significant (p_adj<0.05) change in the percent of the parent population that expresses the marker are noted (increase=pink, decrease=green). (D) Difference in 6-month ZIKV NT_{80} between individuals with or without evidence of prior DENV infection at index visit (Wilcoxon Rank Sum test). Individuals from our sub-cohort are colored black and individuals from the larger REDSIII cohort are colored grey. Scatterplots showing 6-month ZIKV NT_{80} titers versus the frequency of (E) ASC B cells or (F) non-naïve CD8+ T cells co-expressing HLA-DR and CD38 at the acute timepoint (Spearman's correlation). A-C: N=25 participants; E-F: N=14 participants.

ZIKV-infected

Participant #	Age	Sex	Pre-IgM at index visit	DENV exposed	>=3 symptoms at acute visit	Maximum ZIKV NT80 titer	6mo ZIKV NT80 titer
1	52	Male	Y	Y	TRUE	355.1	54.3
2	24	Male	N	N	FALSE	84.4	0
3	22	Male	N	Y	TRUE	9719.1	2894.4
4	62	Male	Y	Y	FALSE	1069.7	217.1
5	43	Male	Y	N	FALSE	NA	NA
6	54	Male	N	Y	FALSE	13952.6	419.9
7	51	Male	Y	Y	FALSE	2782.7	221.7
8	46	Male	Y	Y	TRUE	688	21.6
9	49	Male	N	N	FALSE	1474.1	71.8
10	37	Male	Y	Y	FALSE	37872.3	6285.7
11	46	Male	Y	Y	TRUE	3086.1	100.2
12	36	Female	N	Y	TRUE	4543	202.9
13	43	Male	Y	Y	TRUE	3405.1	1483.7
14	42	Male	Y	Y	TRUE	2238.7	134.8
15	43	Female	N	N	Unknown	1153	77.1
16	28	Female	N	N	Unknown	NA	NA
17	27	Female	Y	N	TRUE	NA	NA
18	53	Male	N	N	FALSE	1198.7	NA
19	25	Female	Y	Y	FALSE	NA	NA
20	71	Male	Y	Y	FALSE	2091.4	440.9
21	67	Male	Y	Y	FALSE	3752.8	237.6
22	24	Male	Y	Y	FALSE	2206.1	2660.2
23	56	Female	Y	Y	TRUE	9412.2	1278.7
24	44	Male	Y	Y	FALSE	23236.4	2828.5
25	21	Female	Y	Y	TRUE	1490.7	779.1

ZIKV-uninfected

Participant #	Age	Sex
26	49	Male
27	32	Female
28	51	Female
29	60	Male
30	53	Male
31	58	Male
32	42	Female
33	22	Male
34	32	Female
35	20	Male
36	40	Male
37	54	Male
38	44	Male
39	20	Male

Table S1. Study participant clinical characteristics (related to: Figure 1).

Cell type	Ki-67	HLA-DR	CD38	CD69	CD71	CD86	CD16	CD40	ICOS	CTLA-4	TIGIT	PD-1	PD-L1	Granzyme B	Tbet	Eomesodermin	Helios
CD14+ monocytes	x			x	x	x	x	x				x	x		x		
CD14-CD16+ monocytes	x			x	x	x		x				x			x		
cDCs	x	x		x	x	x		x				x			x		
pDCs	x		x		x	x						x					
CD56dim NK cells	x	x	x	x	x	x					x	x		x	x	x	
CD56bright NK cells	x	x	x	x	x	x	x				x	x		x	x	x	
Basophils	x				x												
CD8: non-naïve	x	x	x	x	x			x	x	x	x	x	x	x	x	x	x
CD4: Tregs	x	x	x	x					x	x	x	x					x
CD4: non-naïve					x												
CD4 non-naïve: Th1	x	x	x						x		x	x	x	x			x
CD4 non-naïve: Th2	x	x	x						x		x	x	x				x
CD4 non-naïve: Tfh	x	x	x						x			x					
CD4 non-naïve: Th17	x	x	x								x	x					
CD4 non-naïve: Tctl	x	x	x						x		x	x	x	x			x
Vd2+ gd T cells	x	x	x	x							x	x	x	x	x	x	x
Vd2- gd T cells	x	x	x	x							x	x	x	x	x	x	x
Total B cells																	
B cells: transitional	x		x	x	x	x		x				x			x		
B cells: naïve	x		x	x	x	x		x				x			x		
B cells: CD27-IgD-	x		x	x	x	x		x				x			x		
B cells: plasmablast	x		x	x	x	x		x				x			x		
B cells: mem IgD+IgM+	x		x	x	x	x		x				x			x		
B cells: mem IgD-IgM+	x		x	x	x	x		x				x			x		
B cells: mem IgD-IgM-	x		x	x	x	x		x				x			x		

Cell type	CD25	FCRL5	CD21	CD127	CCR4	CCR6	CXCR5	CX3CR1	BDCA1	CD4	CD11b	CD27	CCR7	CD24	total
CD14+ monocytes		x						x	x	x					13
CD14-CD16+ monocytes		x						x	x	x					11
cDCs									x		x				10
pDCs															5
CD56dim NK cells		x		x								x			14
CD56bright NK cells		x		x								x			15
Basophils															2
CD8: non-naïve	x	x		x	x	x		x				x	x		23
CD4: Tregs				x	x	x						x	x		14
CD4: non-naïve		x													2
CD4 non-naïve: Th1	x			x				x				x	x		14
CD4 non-naïve: Th2	x			x								x	x		12
CD4 non-naïve: Tfh	x			x								x	x		9
CD4 non-naïve: Th17	x			x								x	x		9
CD4 non-naïve: Tctl	x			x				x				x	x		14
Vd2+ gd T cells	x			x	x	x		x				x	x		18
Vd2- gd T cells	x			x	x	x		x				x	x		18
Total B cells							x								1
B cells: transitional		x	x						x						11
B cells: naïve		x	x						x					x	12
B cells: CD27-IgD-		x	x						x					x	12
B cells: plasmablast		x	x						x						11
B cells: mem IgD+IgM+		x	x						x					x	12
B cells: mem IgD-IgM+		x	x						x					x	12
B cells: mem IgD-IgM-		x	x						x					x	12
														SUM:	286

Table S2. Summary of phenotypic markers assessed on each cell type for manual gating analysis (related to: Figure 1).
